# HPLC and high-throughput sequencing revealed higher tea-leaves quality, soil fertility and microbial community diversity in ancient tea plantations: compared with modern tea plantations

**DOI:** 10.1186/s12870-022-03633-6

**Published:** 2022-05-12

**Authors:** Guangrong Yang, Dapeng Zhou, Renyuan Wan, Conglian Wang, Jin Xie, Cunqiang Ma, Yongmei Li

**Affiliations:** 1 College of Tea, Yunnan Agricultural University, Kunming, 650201 Yunnan China; 2 College of Horticulture and Forestry, Huazhong Agricultural University, Wuhan, 430070 Hubei China; 3 College of Resources and Environment, Yunnan Agricultural University, Kunming, 650201 Yunnan China

**Keywords:** Ancient tea plantation, Amino acids, Phenolic components, Soil fertility, Microbial community structure

## Abstract

**Background:**

Ancient tea plantations with an age over 100 years still reserved at Mengku Town in Lincang Region of Yunan Province, China. However, the characteristic of soil chemicophysical properties and microbial ecosystem in the ancient tea plantations and their correlation with tea-leaves chemical components remained unclear. Tea-leaves chemical components including free amino acids, phenolic compounds and purine alkaloids collected from modern and ancient tea plantations in five geographic sites (i.e. Bingdao, Baqishan, Banuo, Dongguo and Jiulong) were determined by high performance liquid chromatography (HPLC), while their soil microbial community structure was analyzed by high-throughput sequencing, respectively. Additionally, soil microbial quantity and chemicophysical properties including pH, cation exchange capacity (CEC), soil organic matter (SOM), soil organic carbon (SOC), total nitrogen (TN), total phosphorus (TP), total potassium (TK), alkali-hydrolyzable nitrogen (AN), available phosphorous (AP) and available potassium (AK) were determined in modern and ancient tea plantations.

**Results:**

Tea-leaves chemical components, soil chemicophysical properties and microbial community structures including bacterial and fungal community abundance and diversity evaluated by Chao 1 and Shannon varied with geographic location and tea plantation type. Ancient tea plantations were observed to possess significantly (*P* < 0.05) higher free amino acids, gallic acid, caffeine and epigallocatechin (EGC) in tea-leaves, as well as soil fertility. The bacterial community structure kept stable, while fungal community abundance and diversity significantly (*P* < 0.05) increased in ancient tea plantation because of higher soil fertility and lower pH. The long-term plantation in natural cultivation way might significantly (*P* < 0.05) improve the abundances of *Nitrospirota*, *Methylomirabilota*, *Ascomycota* and *Mortierellomycota* phyla.

**Conclusions:**

Due to the natural cultivation way, the ancient tea plantations still maintained relatively higher soil fertility and soil microbial ecosystem, which contributed to the sustainable development of tea-leaves with higher quality.

**Supplementary Information:**

The online version contains supplementary material available at 10.1186/s12870-022-03633-6.

## Background

Tea (*Camellia sinensis* L.) has become one of the most popular and widely consumed beverages in the world because of its multiple health benefits [1–3]. Southwest China including Yunnan, Guizhou and Sichuan Province has been regarded as the origin center, which is now widely cultivated all over the world for approximately 5000 years [4–6]. Until now, Lincang, Puer and Xishuangbanna Regions in Yunnan Province, China, still remained abundant ancient tea trees of wild, transitional and artificial culture types, respectively [7, 8]. In particular, the fresh tea-leaves collected from the artificial culture trees with an age over 100 years in ancient tea plantations could be processed for Pu-erh tea, black tea and white tea [8].

Processing technology, tea cultivars, production places and harvest seasons significantly impacted phenolic compounds including catechins (a subgroup of flavan-3-ols), phenolic acids, flavonoids and anthocyanins, purine alkaloids, free amino acids like L-theathine, L-glutamate and L-arginine, and flavoalkaloids [9–11]. Additionally, compared with the ecological tea-leaves collected from modern tea plantations, white teas from the ancient tea plantations have been confirmed with higher ameliorative effect on kidney damage in diabetic mice via Sirtuins 1/AMP-activated protein kinase (SIRT1/AMPK) pathway, while Pu-erh teas from ancient tea plantations are discovered to possess higher contents of free amino acids, fatty acids, phenolic acids, nucleosides and nucleobases, but lower contents of flavonoids and caffeine congeners [12, 13]. Generally, the fresh tea-leaves from ancient tea plantations showed relatively higher contents of tea polyphenols, total catechins, free amino acids, gallic acid, soluble sugars and caffeine, but significantly (*P* < 0.05) lower polyphenols/amino acids ratio and ester catechins content, especially epigallocatechin gallate (EGCG). Due to the lower yields, much more nutrients absorbed by deeper roots and greater chemical profile variations, the tea-leaves collected and processed from ancient tea plantations are widely recognized to have better quality and preserving value with a higher price [14].

The 15 rare earth elements contributed to the identification of ancient tea-leaves, which should be attributed to the soil nutrients and ecological system in ancient tea plantations [15]. As the supporter for survival and growth of tea trees, soil nutrients impacted the output and quality of tea-leaves, as well as microbial community composition [16]. In addition, the microbial including bacterial and fungal communities in rhizospheric soil play vital roles in ecological processes, such as soil organic matter (SOM) decomposition, soil nutrient cycling, and soil structure formation and stability [17, 18]. Previous studies have indicated the variation of fungal community in rhizosphere soil with plantation age, and found the significant decrease of soil bacterial community in modern tea plantations after 23 years cultivation, which potentially caused the decrease of glomalin-related soil protein and soil organic carbon (SOC) after long-term cultivation [19, 20]. Unfortunately, the distribution and characteristic of tea-leaves quality components, soil chemicophysical properties and microbial ecosystem in the ancient tea plantations remain unclear.

Thus, in this study, we evaluated the quality components of fresh tea-leaves, and soil chemicophysical properties and microbial community through high performance liquid chromatography (HPLC) and high-throughput sequencing in ancient and modern tea plantations from five geographical sites, which are all located at Mengku Town of Shuangjiang autonomous County in Lincang Region of Yunan Province, China. The following hypotheses were proposed: (1) tea-leaves quality components, soil chemical properties and microbial community would vary with the five geographical sites; (2) ancient tea plantation would differ from the modern tea plantation in soil chemical properties and microbial community, which potentially impacts quality composition of tea-leaves; and (3) soil environmental factors would impact bacterial and fungal community abundances and compositions due to the differences in tea plantation age and cultivation ways. Through comparisons, this study will help to elaborate the sustainable development of ancient tea plantations from soil fertility and microbial community structure.

## Materials and methods

### Experimental sites

**Fig. 1:**
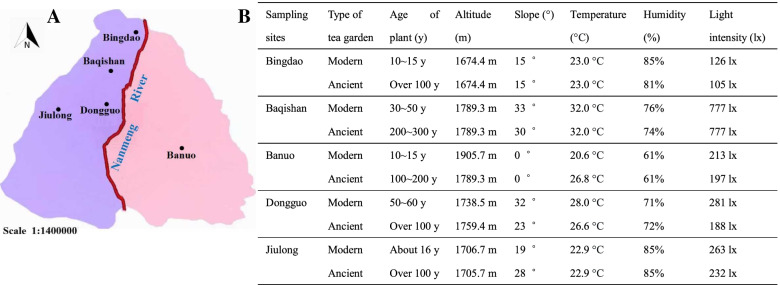
Geographical distribution (**A**) and basic situations (**B**) of five various sampling sites including Bingdao, Banuo, Baqishan, Dongguo and Jiulong. Note: The altitude, slope, temperature, humidity and light intensity were determined by UG903 GPS position indicator (Jiahua Co. Ltd., Suzhou, Jiangsu, China), XW PD001 Digital Level (Xingwangyuda Co. Ltd., Beijing, China) and Vantage Pro 2 Automatic Meteorological Station (Davis Instruments, San Francisco, CA, USA), respectively. These results indicated the similarity of altitude, slope, temperature, humidity and light intensity between modern and ancient tea gardens of each sampling site

The study is performed at representative tea planting area that Mengku Town of Shuangjiang Autonomous County in Lincang Region of Yunan Province, which belongs to the subtropical mountain monsoon climate. The annual average temperature is 18 °C, and the annual average precipitation is from 1700 mm to 1900 mm. The soil type is mainly laterite and yellow loam with the sand grains from granite sandy conglomerate and purple shale. Five sampling sites located at Mengku Town including Bingdao, Banuo, Baqishan, Dongguo and Jiulong all grow ancient tea plantations with an age over 100 years and modern tea plantations with an age no more than 60 years, respectively, which are present in Fig. 1. All tea plantations are grown a single cultivar of tea plant (*Camellia sinesis* var. *assamica* cv. Mengku Dayecha) identified by Yongmei Li and relevant experts, and cultivated in accordance with the requirement of ecological tea plantation that there were no farming, no fertilizers and no pesticides throughout the whole growing period.

### Soil collection and treatment

The fallen leaves, weeds and stones on the surface of the soils from five subplots (0.5 × 0.5 m) in every plot (2 × 2 m) were clean up for the soil sampling collection. The soil samples were collected at the same cleared position. Sterile containers were used to store five soil samples at the 0–20 cm depth from five subplots in every plot, and these soil samples were blended into a composite soil sample. The field-moist clods (< 5 mm) underwent cool-drying at 4 °C until the gravimetric water concentration of about 80 g kg^− 1^ were separated through different sieve mesh including 1 mm, 0.25 mm and 0.15 mm for the determinations of soil physicochemical properties. The field-moist clods collected at 0–4 °C and − 86 °C in aseptic condition were prepared for the determination of microbiological quantification by culture medium and microbial community diversity by high-throughput sequencing, respectively.

### Tea-leaves sample collection

The spring fresh tea-leaves with one bud and two leaves from different tea plantations were collected at the same period with reference to the method of Liu et al. (2020) [21]. The fresh tea-leaves were fixated by a 800 W microwave oven for 60–80 s, and then dried by sunlight until the moisture content below 6.0% by dry weight. The dried tea-leaves were maintained at − 20 °C for the chemical analysis, including tea polyphenols, free amino acids, 6 catechins, caffeine and gallic acid.

### Determinations of main chemical components in tea-leaves

Moisture content and water extracts content were developed according to the national standard methods established by China National Institute of Standardization. Content of tea polyphenols was determined by a UV-1800PC ultraviolet-visible (UV-Vis) spectrophotometer (AOE Instruments, Shanghai, China) using Foline-Ciocalteu assay on 765 nm with gallic acid as the standard [22]. Total content of free amino acids was determined on 570 nm by the UV-Vis spectrophotometer using ninhydrin assay [22].

Six Catechins including catechin (C), epicatechin (EC), epigallocatechin (EGC), epigallocatechin gallate (ECG), gallocatechin gallate (GCG) and epigallocatechin gallate (EGCG), caffeine and gallic acid contents were determined by high-performance liquid chromatography (HPLC) through an Agilent 1200 series HPLC system (Agilent Technologies, Santa Clara, CA, USA) composed of diode array detector, infinity binary pump, integrated vacuum degasser, autosampler and thermostated column compartment and TSK gel ODS-80TM chromatogram column (250 mm × 4.6 mm, 5 μm; Tosoh Corporation, Tokyo, Japan) [23]. Solvent A (0.05 mol L^− 1^ phosphoric acid water solution and 5% acetonitrile) and solvent B (0.05 mol L^− 1^ phosphoric acid water solution and 80% acetonitrile) were prepared for HPLC separation. The gradient was programmed as follows:from 0 to 30 min, solvent A was decreased from 91 to 47%, solvent B was increased from 9 to 53% with a flow rate of 0.7 mL min^− 1^.The column temperature and detection wavelength were set at 30 °C and 280 nm, respectively. Additionally, the injection volume was 5 μL. Quantitative analysis was carried out based on the calibration curves of 8 standards. Quantitative analysis was carried out based on the calibration curves of 8 standards. Each tea-leaves sample was determined with 5 replications.

### Determinations of soil physicochemical properties

The soil pH value was measured by glass recombination electrode method in soil: water solution =1:2.5 (w/w). The cation exchange capacity (CEC) content was determined by ammonium acetate exchange extraction-distillation method. The soil organic carbon (SOC) content was determined by an UV-Vis spectrophotometer method through potassium dichromate with glucose as the standard. The soil organic matter (SOM) content was measured by potassium dichchromate capacity method-external heating method. The total nitrogen (TN) content was measured by Kjeldahl determination after digestion of concentrated sulfuric acid and hydrogen peroxide. The total phosphorous (TP) content was determined by molybdenum antimony anti colorimetric method after digestion of concentrated sulfuric acid and hydrogen peroxide. The total potassium (TK) content was determined by flame photometry after the digestion. The alkali-hydrolyzable nitrogen (AN) content was measured using alkalescent diffusion method of 0.5 mol L^− 1^ NaOH solution. The available phosphorous (AP) content was determined through phosphomolybdate blue spectrophotometry method after the extract of 0.5 mol L^− 1^ NaHCO_3_ solution. The available potassium (AK) content was measured by flame photometry after the extract of ammonium acetate. Each soil sample was determined with 5 replications.

### Determinations of microbial quantity in soil

The microbial quantity including fungi, bacteria and actinomycetes were determined by dilution coating method through the relevant culture mediums, and the colony forming units (CFU) were calculated by per gram of dried soil [24]. Beef extract peptone medium was carried out to measure bacterial quantity after 3 days of cultivation at 28 °C, while fungi number was measured through Martin’s rose Bengal medium after 5 days of cultivation at 28 °C. And the actinomycetes in soil were determined by Gao’s No.1 medium after 7 days of cultivation at 28 °C.

### Soil bacterial and fungal community structure analyses

The 0.5 g of field-moist clods was used to extract DNA through SDS lysis buffer - guanidinium isothiocyanate- polyethylene glycol (SDS-GITC-PEG) method [25]. The extracts were evaluated in terms of both quantity and quality through a NanoDrop ND-2000 spectrophotometer (NanoDrop, Wilmington, DE, USA). The primers 338F (5′-ACTCCTACGGGAGGCAG-3′) and 806R (5′-GGACTACHVGGGTWTCTAAT-3′) were selected to amplify the target V4 hypervariable regions of bacterial 16S rRNA genes for bacterial community structure analysis, while the primers ITS3F (5′-GCATCGATGAAGAACGCAGC-3′) and ITS4R (5′-TCCTCCGCTTATTGATATGC-3′) were used for the amplification of the target V3-V4 hypervariable regions of fungal 18S rRNA genes for fungal community structure analysis [26, 27]. The final volume of 20 μL included 4 μL of 5 x TransStart FastPfu buffer, 2 μL of dNTPs (2.5 mM), 0.5 μL of Taq polymerase, 0.8 μL of each primer (5 μM), 1.0 ng of containing template DNA and 0.4 μL of TransStart FastPfu DNA polymerase were used to implement amplifications. The PCR reaction procedure was performed by ABI 9700 PCR amplification (Applied Biosystems, Waltham, MA, USA) as follows. Pre-degeneration at 95 °C for 2 min, degeneration at 98 °C for 10 s, annealing at 62 °C for 30 s, extension at 68 °C for 30 s, with 27 cycles, extension at 68 °C for 10 min. PCR amplified products were detected and purified via 2% (m/v) Agarose gel electrophoresis. An Illumina Miseq PE300 sequencer (Illumina, San Diego, CA, USA) implemented the paired-end sequencing of PCR amplification.

The fastp software was adopted for the quality control of original sequencing sequence. The FLASH software was carried out for DNA fragment assembly, through which sequences of < 50 bp and the reads with ambiguous bases, could be removed. The same operational taxonomic units (OTUs) were clustered by the sequences at the 97% identity threshold using UPARSE software. An 70% threshold was adopted to determine the taxonomic assignments of the OTUs through the Silva and Unite database. At OTUs level, the soil microbial community diversity, including the Chao 1 estimator and Shannon diversity were calculated using MOTHUR software [26].

### Statistical analyses

The raw data of tea-leaves quality components, soil environmental factors and microbial community composition in modern and ancient tea plantations of five sampling sites are present in Additional file 1. The independent-samples T-test was carried out to compare tea-leaves chemical components, soil chemicophysical properties, and microbial community structure between ancient and modern tea plantations using SPSS 20.0 for Windows (IBM, Armonk, NY, USA). Principal coordinates analysis (PCoA) and non-metric multidimensional scaling (NMDS) were employed to evaluate the differences of soil microbial community composition according to Bray-Curtis distances between two types of tea plantations using Canoco 4.5 software (Microcomputer Power, Ithaca, NY, USA). The interactions among tea-leaves chemical components, soil environmental factors and microbial community composition in tea plantation were confirmed through the bivariate correlation analysis of SPSS 20.0 for Windows (IBM, Armonk, NY, USA) and the redundancy analysis (RDA) of Origin 9.0 software (OriginLab, Hampton, MA, USA), respectively.

## Results and discussion

### Tea-leaves quality components variations with geographic site and tea plantation type

Except that the significant (*P* < 0.05) differences of chemical components were found in tea-leaves among five various geographic sites (Additional file 2: Table S1), the independent-samples T-test analysis indicated the definite differences of tea-leaves chemical components between modern and ancient tea plantations (Table 1), such as free amino acids and catechins, which revealed chemical components variations caused by geographical distribution and plantation type. In all sampling sites (Additional file 2: Table S1), tea-leaves collected from Bingdao possessed the highest levels of chemical components, including free amino acids, caffeine, gallic acid, GCG and ECG. Conversely, the tea-leaves from Baqishan had highest polyphenols/amino acids ratio value, and relatively higher contents of tea polyphenols, C and EGC, which was consistent with the report by Wang et al. (2018) [28], who discovered the differences of metabolic components in raw Pu-erh teas from various mountains.

**Table 1:** Chemical indicators of tea-leaves in modern and ancient tea plantations, respectively

Item	Modern tea plantations		Ancient tea plantations	
	Mean (*n* = 5*5)	Rang	Mean (*n* = 5*5)	Rang
Water extraction (%)	49.74 ± 2.65	48.65 ~ 50.84	50.82 ± 2.09	49.96 ~ 51.69
Free amino acids (%)	2.81 ± 0.36	2.65 ~ 2.96	3.43 ± 0.30***	3.31 ~ 3.55
Tea polyphenols (%)	29.47 ± 1.44	28.87 ~ 30.06	30.83 ± 1.77**	30.10 ~ 31.56
Polyphenols/Amino acids ratio	10.74 ± 2.03	9.90 ~ 11.58	9.08 ± 1.23***	8.57 ~ 9.59
Caffeine (mg g^− 1^)	40.21 ± 3.93	38.59 ~ 41.83	42.78 ± 3.33*	41.40 ~ 44.15
Gallic acid (mg g^−1^)	0.09 ± 0.18	0.01 ~ 0.16	0.56 ± 0.11***	0.51 ~ 0.60
C (mg g^−1^)	15.74 ± 4.94	13.70 ~ 17.78	13.85 ± 5.45	11.60 ~ 16.11
EC (mg g^−1^)	28.34 ± 7.60	25.20 ~ 31.47	30.10 ± 10.33	25.84 ~ 34.36
EGC (mg g^−1^)	18.20 ± 5.45	15.95 ~ 20.44	30.47 ± 3.52***	29.02 ~ 31.93
ECG (mg g^−1^)	85.66 ± 19.44	77.64 ~ 93.69	75.04 ± 20.66	66.51 ~ 83.57
GCG (mg g^−1^)	5.45 ± 1.50	4.83 ~ 6.07	4.35 ± 1.78*	3.61 ~ 5.08
EGCG (mg g^−1^)	53.40 ± 6.28	50.81 ~ 55.99	44.92 ± 4.70***	42.98 ~ 46.86
Total catechins (mg g^−1^)	200.79 ± 35.87	191.98 ~ 221.60	198.74 ± 37.51	183.25 ~ 214.22
Non-ester catechins (mg g^−1^)	62.28 ± 16.70	55.38 ~ 69.17	74.43 ± 15.51*	68.02 ~ 80.83
Ester catechins (mg g^−1^)	144.51 ± 22.38	135.28 ~ 153.75	124.31 ± 22.91**	114.85 ~ 133.76
Non-ester/ Ester catechins ratio	0.43 ± 0.09	0.39 ~ 0.46	0.60 ± 0.05***	0.57 ~ 0.62

Note: Polyphenols/Amino acids ratio = Tea polyphenols content/ free amino acids content; Total catechins were the summation of C, EC, EGC, ECG, GCG and EGCG contents; Non-ester catechins were the summation of C, EC and EGC contents; Ester catechins were the summation of ECG, GCG and EGCG contents; Non-ester/ Ester catechins ratio = Non-ester catechins content/Ester catechins content

And all data were present by mean value ± standard deviation. The range was the mean value at 95% confidence coefficient. Significance difference levels: * *P* < 0.05; ** *P* < 0.01 and *** *P* < 0.001 determined by the independent-samples T-test using SPSS 20.0 for Windows

Among 16 chemical indicators, 11 chemical indicators showed significant (*P* < 0.05), highly significant (*P* < 0.01) or extremely significant (*P* < 0.001) differences, including tea polyphenols, free amino acids, caffeine, gallic acid, EGC, GCG and EGCG, particularly polyphenols/amino acids ratio and non-ester/ ester catechins ratio with the extremely significant (*P* < 0.001) differences between modern and ancient tea plantations. Compared with modern tea plantations, ancient tea plantations were confirmed to possess higher contents of free amino acids, tea polyphenols, caffeine, gallic acid, EGC and total non-ester catechins, but had significantly (*P* < 0.05, *P* < 0.01 or *P* < 0.001) lower contents of GCG, EGCG and total ester catechins, which was consistent with the report by Cheng et al. (2018) [29] in EGCG content, while Ge et al. (2021) [13] discovered the catechins levels decreased after long-term plantation for about 1000 years. The extremely significantly (*P* < 0.001) lower polyphenols/amino acids ratio and higher non-ester/ ester catechins ratio in fresh tea-leaves from ancient tea plantations indicated the remarkable difference in chemical compositions distribution of free amino acids, catechins and phenolic compounds between modern and ancient tea plantations. Generally, the average contents of free amino acids, gallic acid and EGC in tea-leaves from ancient tea plantations were 1.22, 6.22 and 1.67 times higher than that from modern tea plantations, which should be attributed to the physiological metabolisms of tea plant, such as amino acid metabolism, caffeine metabolism and phenolic compounds metabolism associated with the soil environment and ecological system of tea plantation. Due to the nitrogen metabolism in tea plant, several new flavoalkaloids were found in green tea-leaves collected from the ancient tea plantation [29].

### Soil chemicophysical properties and microbial quantity in various tea plantations

Soil chemicophysical properties and microbial quantity varied significantly (*P* < 0.05) with five geographical sites and two types of tea plantations (Table 2 and Additional file 2: Table S2). The highest levels of SOM, AN and AP were observed in Bingdao site, while Jiulong site possessed the relatively lower soil fertility (Additional file 2: Table S2). Conversely, the soil pH (ranging from 4.18 to 4.77) kept relative stable among five geographical sites. About microbial quantity, there were no significant (*P* ≥ 0.05) differences between two types of tea plantations on the whole. However, fungi population was extremely significantly (*P* < 0.001) increased in ancient tea plantations (11.25 ± 3.54 × 10^3^ CFU g^− 1^) compared with the modern tea plantations (8.22 ± 1.63 × 10^3^ CFU g^− 1^), which indicated that the long-term plantation age or natural cultivation way improved fungi community quantity in ancient tea plantations. However, bacteria and actinomycetes kept relatively stable with no significant (*P* ≥ 0.05) variations in modern and ancient tea plantations.

**Table 2:** Soil physical properties and microbial population in modern and ancient tea plantations, respectively

Item		Modern tea plantations		Ancient tea plantations	
		Mean (*n* = 5*5)	Rang	Mean (*n* = 5*5)	Rang
**Soil physical and chemical properties**	pH value	4.59 ± 0.13	4.53 ~ 4.64	4.47 ± 0.21*	4.38 ~ 4.55
	CEC (cmol kg^−1^)	9.13 ± 3.34	7.75 ~ 10.51	12.15 ± 2.84**	10.98 ~ 13.33
	SOC (g kg^−1^)	38.67 ± 12.56	33.49 ~ 43.86	51.31 ± 8.29***	47.89 ~ 54.73
	SOM (g kg^−1^)	65.93 ± 21.88	56.90 ~ 74.96	88.66 ± 13.20***	83.21 ~ 94.11
	TN (g kg^−1^)	1.19 ± 0.49	0.98 ~ 1.39	1.68 ± 0.45**	1.50 ~ 1.87
	TP (g kg^−1^)	0.257 ± 0.122	0.206 ~ 0.307	0.502 ± 0.162***	0.435 ~ 0.569
	TK (g kg^−1^)	6.59 ± 1.19	6.10 ~ 7.08	5.21 ± 2.67*	4.11 ~ 6.31
	AN (mg kg^−1^)	100.55 ± 26.04	89.80 ~ 111.29	128.88 ± 36.09**	113.99 ~ 143.78
	AP (mg kg^−1^)	10.64 ± 3.41	9.23 ~ 12.05	18.19 ± 2.49***	17.16 ~ 19.22
	AK (mg kg^−1^)	132.76 ± 14.57	126.74 ~ 138.78	151.49 ± 41.24*	134.46 ~ 168.51
**Soil microbial population**	Bacteria (×10^6^CFU g^−1^)	20.88 ± 7.72	17.70 ~ 24.07	20.22 ± 3.86	18.63 ~ 21.81
	Fungi (×10^3^CFU g^−1^)	8.22 ± 1.63	7.54 ~ 8.89	11.25 ± 3.54***	9.79 ~ 12.71
	Actinomycetes (×10^7^CFU g^−1^)	9.77 ± 7.15	6.82 ~ 12.72	10.18 ± 4.75	8.22 ~ 12.15

*CEC* Cation exchange capacity, *SOC* Soil organic carbon, *SOM* Soil organic matter, *TN* Total nitrogen, *TP* Total phosphorus, *TK* Total potassium, *AN* Alkali-hydrolyzable nitrogen, *AP* Available phosphorus, *AK* Available potassium

All data were present by mean value ± SD. The range was the mean value at 95% confidence coefficient. Significance difference levels: * *P* < 0.05; ** *P* < 0.01 and *** *P* < 0.001 determined by the independent-samples T-test using SPSS 20.0 for Windows

Compared with the modern tea plantations, due to the micro-climate environment, low intensity production mode and less disturbance in cultivation management, the ancient tea plantations contained significantly (*P* < 0.05) higher soil fertility, including SOM, SOC, CEC, TN, TP, AN, AP and AK, but relative lower soil pH and TK. CEC (10.98–13.33 cmol kg^− 1^, range across all the soil samples), SOC (47.89–54.73 g kg^− 1^), SOM (83.21–94.11 g kg^− 1^), TN (1.50–1.87 g kg^− 1^), TP (0.435–0.569 g kg^− 1^), TK (4.11 ~ 6.31 g kg^− 1^), AN (113.99–143.78 mg kg^− 1^), AP (17.16–19.22 mg kg^− 1^) and AK (134.46–168.51 mg kg^− 1^) contents were accumulated in ancient tea plantations (Table 2). The soil in modern tea plantations was high acidification after 60-years plantation [30]. Despite that the soil pH value (ranging from 4.38 to 4.55) in ancient tea plantations was significantly (*P* < 0.05) lower than that in modern tea plantations, it was still optimum pH condition for the growth of tea trees [31]. The higher CEC, SOM and SOC in ancient tea plantations delayed soil acidification trend through relevant microbial metabolic activity [32].

In spite of a long-term plantation age over 100 years, the ancient tea plantations still kept relatively higher soil microbial metabolic activities, particularly fungi communities with an extremely significant (*P* < 0.001) increase, which should be attributed to the natural cultivation way of ancient tea plantations [33]. Generally, the ancient tea plantations were cultivated close to tall trees and sheltered by virgin forests that contributed to the accumulation of SOM and SOC due to the higher litter derived from tea plant and other plants [34]. The higher nitrogen (N), phosphorus (P) and potassium (K) levels, particularly AN, AP and AK, ensure the sustainable development for the healthy growth of tea trees and the production of fresh tea-leaves in the ancient tea plantations. Except the TK with a relatively higher loss that was translated into AK under the effect of related microbial communities, the natural cultivation way was conducive to a growth over 100 years in ancient tea plantations because of the higher soil fertility. The higher AN, AP and AK improved theanine and flavonoid biosynthesis activities in tea plant physiology through glutamine synthetase, phenylpropanoid, flavonoid pathways [35, 36], which might contribute to the accumulations of free amino acids, gallic acid, caffeine and EGC in tea-leaves. Additionally, the photosynthetic capacity potentially impacted catechins biosynthesis (such as EGC) in tea plant [37], which might cause the significant (*P* < 0.05) differences of EGC, EGCG and GCG between modern and ancient tea plantations.

### Soil microbial community abundance and diversity in various tea plantations

A total of 6978 OUTs belonging to 30 phyla, 125 classes, 293 orders, 455 families, 856 genera and 1818 species were obtained by analyzing the bacterial communities, while a total of 5525 OUTs belonging to 20 phyla, 68 classes, 170 families, 853 genera and 1408 species were obtained by analyzing the fungal communities in all soil samples through the Illumina high-throughput sequencing, respectively. The values of bacterial coverage estimators ranged from 96.94 to 97.70%, and the values of fungal coverage estimators were all over 99.23% at 97% similarity cutoff, which suggested that the current numbers of the sequence reads were sufficient to reflect the soil bacterial and fungal community diversity (Additional file 2: Table S3).

Bacterial community abundance kept relatively stable among five geographic sites and two types of tea plantations ranging from 3375 OUTs to 4508 OUTs. Conversely, the independent-samples T-test analysis indicated that the fungal community abundance varied significantly (*P* < 0.05) with the geographic sites and tea plantation types. Generally, the ancient tea plantations involved a significant (*P* < 0.05) higher fungal community abundance. Therefore, plantation age enhanced the soil fungal community abundance in the natural cultivation way, while the bacterial community abundance kept stable with slight increases in the ancient tea plantations.

About α-diversity evaluated by Chao 1 and Shannon indices (Additional file 2: Table S3; Table S4), soil bacterial abundance kept stable with no significant (*P* ≥ 0.05) variations among five geographical sites and two types of tea plantations, except that the Chao 1 increased highly significantly (*P* < 0.01) in the ancient tea plantation (3215.36) of Jiulong site compared with the modern tea plantation (2754.08). However, Chao 1 and Shannon indices in soil fungal community diversity varied significantly (*P* < 0.05) with geographical sites and tea plantations type. Generally, α-diversity (i.e Chao 1 and Shannon indices) of fungal communities in ancient tea plantations were highly significantly (*P* < 0.01) or extremely significantly (*P* < 0.001) higher than that in modern tea plantations, respectively. Particularly, among five geographical sites, the ancient tea plantations located at Banuo, Dongguo and Jiulong sites showed significantly (*P* < 0.05) or highly significantly (*P* < 0.01) higher fungal community diversity than modern tea plantations. Compared with bacterial communities remaining stable along with the plantation age and cultivation ways, the fungal communities abundance and α-diversity significantly (*P* < 0.05) increased in the ancient tea plantations, which should be attributed to the long-term plantation age and natural cultivation way.

Previous studies confirmed that bacterial community diversity significantly (*P* < 0.05) decreased after long-term plantation over 23-years in modern tea plantations [19, 30]. However, despite the long-term plantation over 100 years, the bacterial community abundance and diversity kept stable in ancient tea plantations because of the natural cultivation way that maintained relatively higher soil fertility. The significantly (*P* < 0.05) higher fungal abundance and diversity in ancient tea plantations was consistent with the report by Wu et al. (2020) [38], which confirmed that the long-term plantation might improve the fungal community in rhizosphere soil.

### Bacterial community composition in modern and ancient tea plantations

**Fig. 2:**
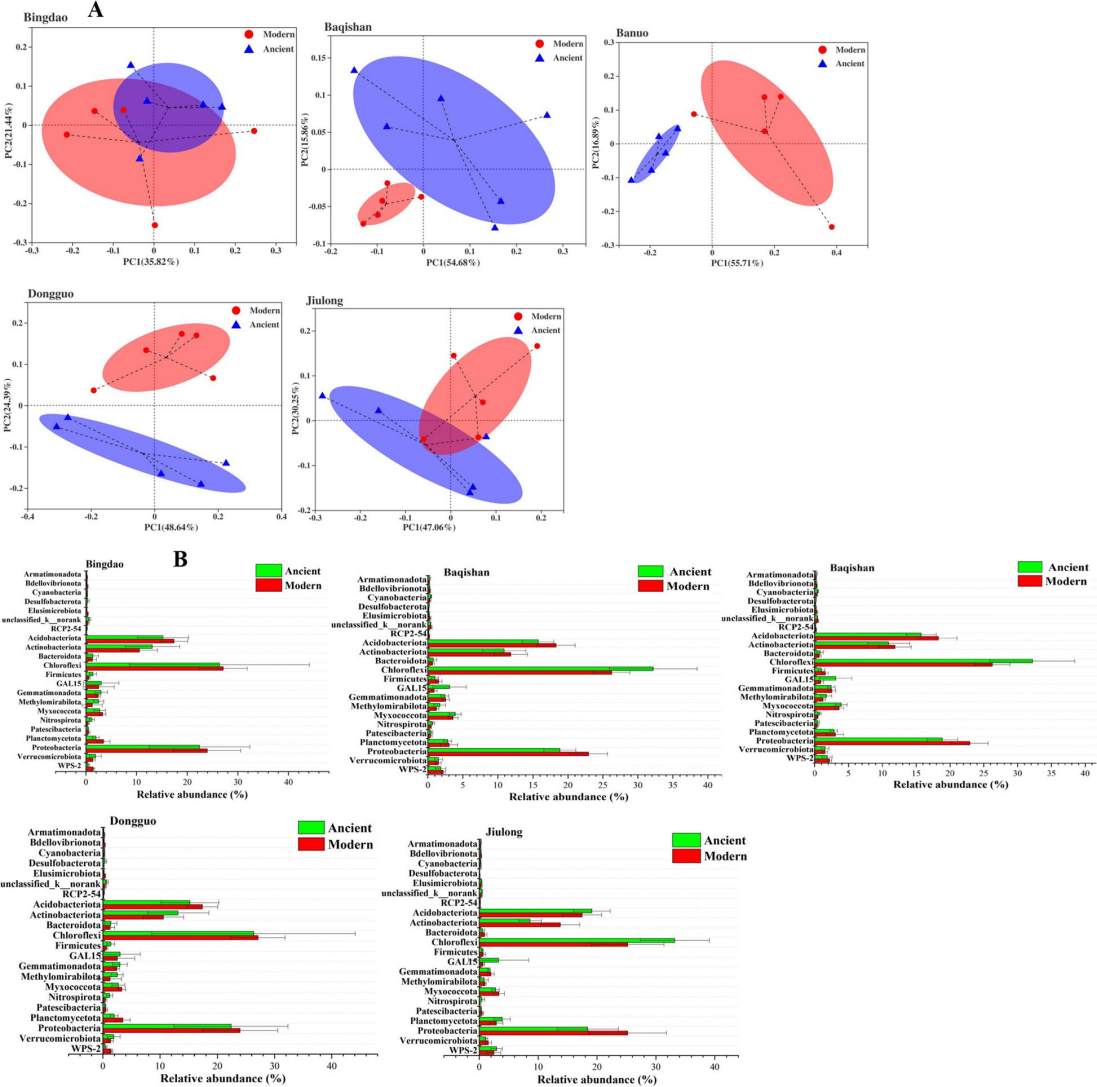
Principal coordinate analysis (PCoA, **A**) showed the remarkable difference of soil bacterial community composition between modern and ancient tea plantations in five sampling sites including Bingdao, Baqishan, Banuo, Dongguo and Jiulong, and their relative abundance differences in the top 22 phyla (**B**) through independent-samples T-test, respectively

Both PCoA (Fig. 2A) and NMDS (Additional file 2: Fig. S1) indicated the remarkable differences of bacterial community composition in soil aggregates between and ancient tea plantations. In the PCoA, the first two principal coordinates together represented 57.26, 70.54, 72.60, 73.03 and 77.31% of the variations by PC1 and PC2 in soil bacterial community composition from two types of tea plantations located at Bingdao, Baqishan, Banuo, Dongguo and Jiulong, respectively, which demonstrated that the bacterial community from same type of tea plantation in five various geographical sites was almost grounded together, while the bacterial community from the ancient tea plantation in the same geographical site were well separated from each other. Additionally, NMDS analysis revealed that the bacterial community composition between modern and ancient tea plantations in the same geographical site was significantly (*P* < 0.05) distinguished.

At the phylum level in soil bacterial community composition (Fig. 2B), *Chloroflexi* (24.94%), *Proteobacteria* (22.39%), *Acidobacteriota* (17.00%) and *Actinobacteriota* (13.15%) were regarded as the dominant groups in ancient tea plantations, which kept relatively stable in modern tea plantations with no significant (*P* ≥ 0.05) variations. Compared to the modern tea plantations, *Proteobacteria* and *Actinobacteriota* showed slight downward trend, while *Chloroflexi* and *Acidobacteriota* showed slight rising tendency in ancient tea plantations. Particularly, due to the long-term plantation and natural cultivation way, the relative abundances of *Nitrospirota* and *Methylomirabilota* significantly (*P* < 0.05) or highly significantly (*P* < 0.01) increased, but the relative abundances of *Bacteroidota*, *Patescibacteria* and *Bdellovibrionota* significantly (*P* < 0.05) or extremely significantly (*P* < 0.001) decreased in the ancient tea plantations (Additional file 2: Table S5).

At the genus level, *c__AD3* (12.01%), *f__Xanthobacteraceae* (5.62%), *o__Acidobacteriales* (4.38%), *o__Subgroup_2* (3.04%), *o__Vicinamibacterales* (2.57%), *Bradyrhizobium* (2.22%), *f__Gemmataceae* (2.07%), *HSB_OF53-F07* (2.05%), *o__IMCC26256* (2.03%) and *c__TK10* (2.01%) were the dominate genera in bacterial community of ancient tea plantations. Particularly, *f__Xanthobacteraceae*, *c__TK10* and *HSB_OF53-F07* significantly (*P* < 0.05) increased in the ancient tea plantations. Additionally, *o__Acidobacteriales* and f*__Acidobacteriaceae_Subgroup_1* might contribute to the stabilization of soil pH in ancient tea plantations. Among the top 50 bacterial genera in relative abundance (Additional file 2: Table S6), compared with the modern tea plantations, 5 genera (i.e. *f__Xanthobacteraceae*, *c__TK10*, HSB_OF53-F07, *o__Rokubacteriales* and *Nitrospira*) significantly (*P* < 0.05) or highly significantly (*P* < 0.01) increased, while 6 genera (i.e. *Sphingomonas*, *Burkholderia-Caballeronia-Paraburkholderia*, *Mycobacterium*, *Haliangium*, *Gemmatimonas* and *Kitasatospora*) significantly (*P* < 0.05) decreased in the ancient tea plantations. Particularly, as the main nitrifying bacteria, the improvement of *Nitrospirota* phylum and *Nitrospira* genus in relative abundance significantly (*P* < 0.05) enhanced the level of AN and soil fertility of ancient tea plantations. Additionally, *HSB_OF53-F07* and *Acidothermus* genera improved the soil carbon metabolism in ancient tea plantations.

The dominant phyla in bacterial community composition of tea plantations were consistent with the studies of Wang et al. (2019) [19] and Zheng et al. (2020) [39], while Chen et al. (2020) [30] discovered that *Proteobacteria*, *Acidobacteriota*, *Actinobacteriota* and *Firmicutes* were dominate phyla in soil of tea plantations. Although *Actinobacteriota* increased toward lower soil pH often [40], it kept stable in relative abundance in ancient tea plantations. As the main soil probiotics, the slight improvement of *Bacillus* genus enhanced the soil fertility and conduced to the sustainable development of ancient tea plantations.

### Fungal community composition in modern and ancient tea plantations

**Fig. 3:**
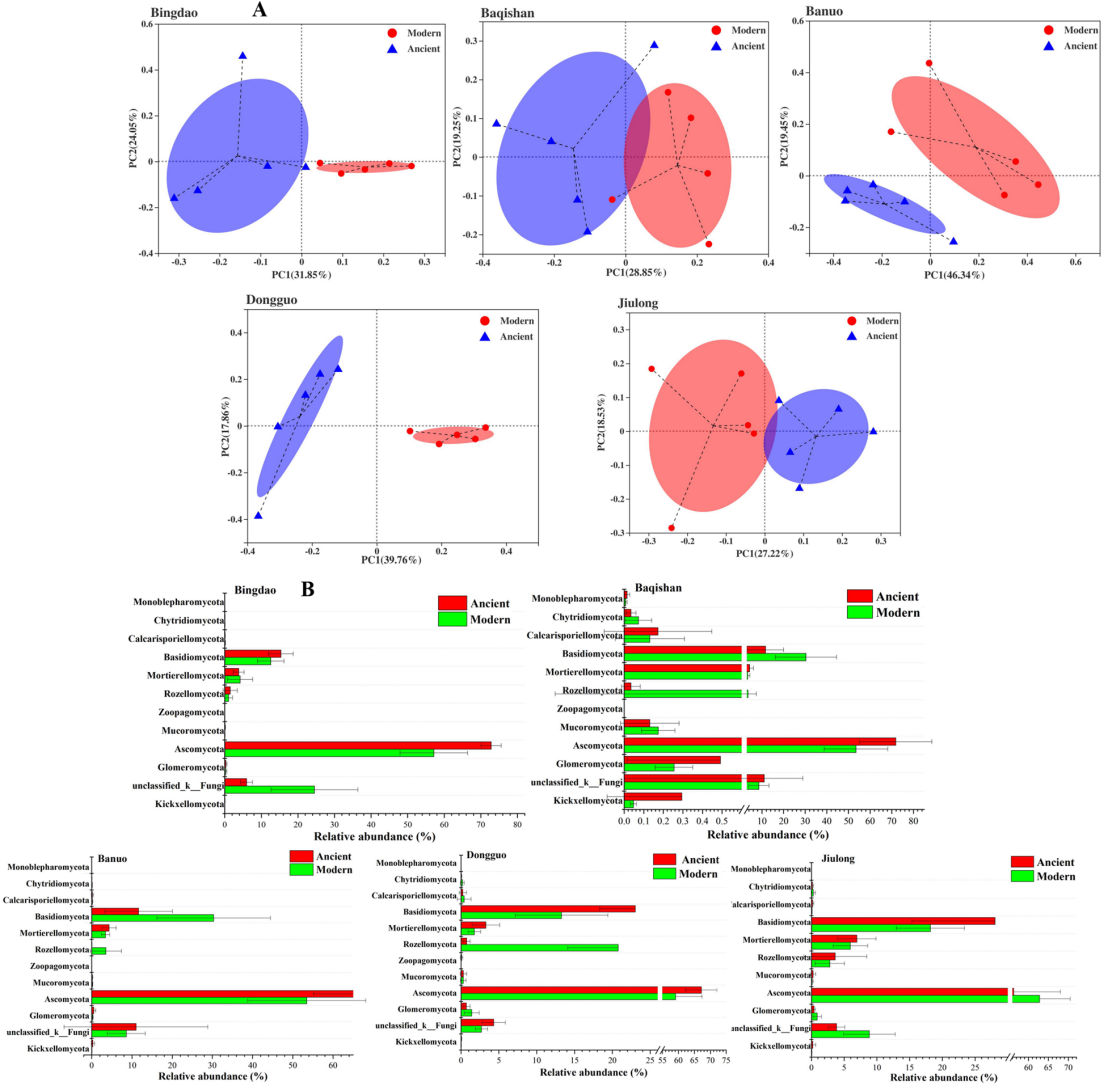
Principal coordinate analysis (PCoA, **A**) showed the remarkable difference of soil fungal community composition between modern and ancient tea plantations in five various sampling sites, and their relative abundance differences in the top 12 phyla (**B**) through independent-samples T-test, respectively

Similar to bacterial community composition, fungal community composition varied significantly (*P* < 0.05) with geographical site and tea plantation types through PCoA (Fig. 3A) and NMDS analysis (Additional file 2: Fig. S2). In the PCoA, the first two principal coordinates together represented 55.90, 48.10, 65.79, 57.62 and 45.75% of the variations by PC1 and PC2 in soil fungal community composition between modern and ancient tea plantations located at Bingdao, Baqishan, Banuo, Dongguo and Jiulong, respectively, which indicated the remarkable differences in soil fungal community composition between modern and ancient tea plantations. The results of PCoA were consistent with NMDS analysis (Additional file 2: Fig. S2), demonstrated the extremely significantly (*P* < 0.001) distinction in fungal community composition between two types of tea plantations in the same geographical site.

At the phylum level in soil fungal community composition (Fig. 3B), *Ascomycota* (65.75%), *Basidiomycota* (19.02%), *Mortierellomycota* (5.08%), *Rozellomycota* (1.61%), *Glomeromycota* (0.39%) and *Unclassified-k-Fungi* (7.57%) were the dominant groups in ancient tea plantations. Except for Jiulong, the relative abundance of *Ascomycota* in the ancient tea plantations of other four sites was significantly (*P* < 0.05) higher than that in modern tea plantations, which occupied from 51.50 to 70.28%. The soil fungal community composition were significant (*P* < 0.05) differences across five sampling sites. For example, the relative abundance of *Rozellomycota* in modern tea plantation of Dongguo site reached 20.77%, but its relative abundance in other soil aggregates was relatively low. Compared to modern tea plantations, *Unclassified-k-Fungi* and *Rozellomycota* significantly (*P* < 0.05) decreased, while *Ascomycota* and *Mortierellomycota* significantly (*P* < 0.05) increased in the ancient tea plantations (Additional file 2: Table S7).

At the genus level, *Saitozyma* (11.17%), *p_*_*Ascomycot*a (11.15%), *Unclassified_k_*_*Fungi* (7.57%), *Mortierella* (5.02%), *Chaetomium* (4.32%), *Penicillium* (3.33%), *o_*_*Chaetothyriales* (3.31%), *c_*_*Eurotiomycetes* (2.89%), *f__Didymellaceae* (2.30%), *f__Clavariaceae* (2.19%) and *Fusarium* (1.82%) were identified as the dominate genera in ancient tea plantations (Additional file 2: Table S8). Compared to the modern tea plantations, the relative abundances of *Saitozyma*, *Metarhizium*, *Mortierella*, *f_*_*Clavariaceae*, *Pseudogymnoascus*, *o__Helotiales*, *Leohumicola*, *Tolypocladium* and *Trichoglossum* significantly (*P* < 0.05), highly significantly (*P* < 0.01) or extremely significantly (*P* < 0.001) increased in the ancient tea plantations. Additionally, 3 genera including *Unclassified_k*__*Fungi*, *p_*_*Rozellomycota* and *Phialocephala* significantly (*P* < 0.05) decreased in ancient tea plantations.

The dominant phyla in fungal community composition were consistent with the report by Wu et al. (2020) [38]. The abundances of *Ascomycota* and *Mortierellomycota* phyla, as well as 9 genera (i.e. *Saitozyma*, *Metarhizium*, *Mortierella*, f__*Clavariaceae*, *Pseudogymnoascus*, o__*Helotiales*, *Leohumicola*, *Tolypocladium* and *Trichoglossum*) significantly (*P* < 0.05) increased with the plantation age in natural cultivation way, which should be attributed to the higher fertility in the ancient tea plantations. The entomopathogenic fungi stay at relatively lower level in ancient tea plantations. For instance, the abundance of *Fusarium* genus showed a slight decrease from 2.86 to 1.82% in the ancient tea plantations.

### Potential impact of soil physical properties in tea plantations

The bivariate correlation analysis was carried out to explore the potential impact of soil physical properties on tea-leaves quality components and microbial community in tea plantations through the relevant Spearman correlation coefficient (Additional file 2: Table S9; Table S10). The soil TN, TP, AN, and AP levels might improve the contents of free amino acids, gallic acid and EGC in tea-leaves with the significantly (*P* < 0.05) or highly significantly (*P* < 0.01) positive correlations (r-value ranging from 0.355 to 0.624), but decreased EGCG content with the highly significantly (*P* < 0.01) negative correlations (r-value = − 0.451, − 0.471 and − 0.611, respectively). Additionally, TK and AK impacted potentially the levels of phenolic components including gallic acid, C, EC, EGC, ECG, GCG and EGCG, due to the significantly (*P* < 0.05) or highly significantly (*P* < 0.01) negative correlations (Additional file 2: Table S9). These results indicated that soil environmental factors, such as CEC, SOC, SOM, nitrogen (N), phosphorus (P) and potassium (K), showed significant (*P* < 0.05) associations with tea-leaves quality components, particularly free amino acids and phenolic components. Generally, SOC, SOM, N and P might contribute to the accumulations of free amino acids, gallic acid and EGC, while the K might impact the biosynthesis of phenolic compounds, including gallic acid, C, EC, EGC, ECG, GCG and EGCG, which generated the chemical differences in tea-leaves between modern and ancient tea plantations.

The soil pH was main environmental factor impacting the microbial abundance and diversity. In the modern tea plantations, the bacterial abundance and diversity decreased significantly (*P* < 0.05) after the long-term plantation due to the continuous acidification in the soil [19, 28]. However, despite the reduction of bacterial abundance with the lower soil pH, the relatively lower soil pH improved the abundances of actinomycetes and fungi in ancient tea plantations. Additionally, the microbial diversity including bacterial and fungal diversity was not sensitive to soil pH (Additional file 2: Table S10). Differed form soil pH, the soil fertility, such as SOC, SOM and TP, might improve fungi abundance determined by dilution coating method. Additionally, the soil P level showed the significantly (*P* < 0.05) or highly significantly (*P* < 0.01) positive correlations with the abundance and diversity of fungi community, and actinomycetes richness, while AK level might improve bacterial community abundance. The N level in soil had limited influence on soil microbial community abundance and diversity with no significant (*P* ≥ 0.05) correlations to bacterial and fungal communities.

### Interactions between soil physical properties and microbial community composition

**Fig. 4:**
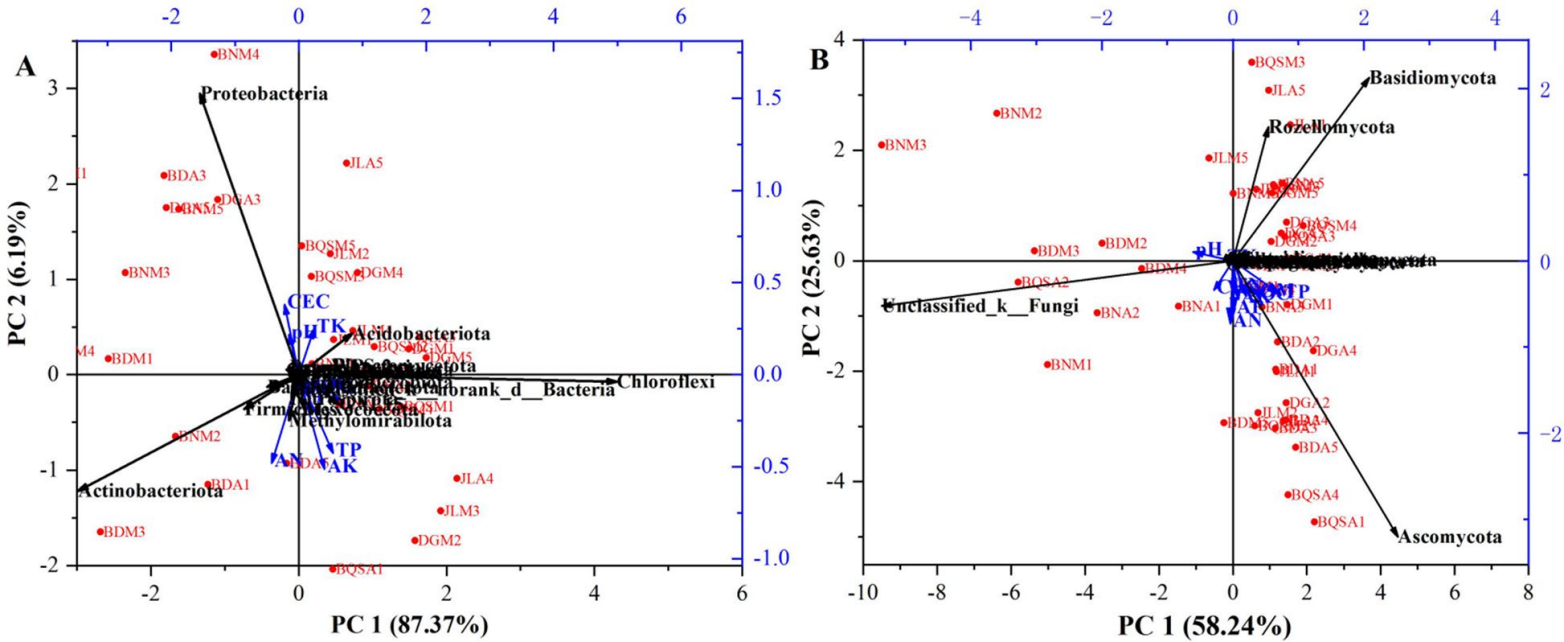
Internal relationships of soil environmental factors to bacterial (**A**) and fungal (**B**) community composition at the phylum level in tea plantation through redundancy analysis (RDA)

The Spearman correlation coefficient determined by the bivariate correlation analysis corresponded highly to the RDA. In this study, the top 22 bacterial phyla (Fig. 4A) and 12 fungal phyla in microbial communities were carried out as the response variables through the RDA to elaborate the impact of soil environmental factors on microbial community composition in tea plantations, respectively. The RDA model accounted for 93.56 and 86.87% of the total variation of soil bacterial and fungal community composition in tea plantations, respectively. AK, AN, TP, CEC and TK were main environmental factors affecting bacterial community composition in turn (Fig. 4A). CEC, TK and soil pH showed significantly (*P* < 0.05) positive correlations with the relative abundances of *Proteobacteria* and *Acidobacteriota*, while the positive correlations of the relative abundances of *Actinobacteria* and *Chloroflexi* to AK, AN, and AP were found in the bacterial community composition (Fig. 4A). Therefore, CEC contributed to the improvement of f__*Xanthobacteraceae*, while TK and soil pH impacted *Sphingomonas* and *Burkholderia-Caballeronia-Paraburkholderia* genera in ancient tea plantations. Additionally, the soil AN, AP and AK showed positive correlations with the abundances of *c__TK10*, *HSB_OF53-F07*, *Mycobacterium* and *Kitasatospora* in ancient tea plantations.

However, for the fungal community composition in tea plantations, TP, AN, AP, pH and AK were principal influence factors. The abundance of *Ascomycota* phylum in fungal community composition showed significantly (*P* < 0.05) positive correlations to soil chemical properties, such as AN, AP, AK, SOC, SOM and TP, but with negative correlation to soil pH, which indicated that the soil chemical properties improved the abundance of *Ascomycota* phylum including *Metarhizium*, *Pseudogymnoascus*, *o__Helotiales*, *Leohumicola*, *Tolypocladium* and *Trichoglossum* genera toward to the lower pH in the ancient tea plantations. Soil pH and TN showed definitely positive corrections to the abundances of *Unclassified_k__Fungi*, *Basidiomycota* and *Rozellomycota*, which revealed that these fungal phyla were sensitive to the soil pH with significant (*P* < 0.05) decreases in ancient tea plantations (Fig. 4B). Generally, the significant (*P* < 0.05) increases of *Ascomycota* phylum as well as *Metarhizium*, *Pseudogymnoascus*, *o__Helotiales*, *Leohumicola*, *Tolypocladium* and *Trichoglossum* genera in the abundances were caused by the higher soil fertility, and lower pH value contributed to the stabilization of soil microbial diversity and abundance in ancient tea plantations.

#### Conclusions

Due to the excellent ecological environment, natural cultivation way and complete community structure, the ancient tea plantations were observed to possess significantly (*P* < 0.05) higher free amino acids, caffeine, gallic acid and EGC contents, but lower GCG and EGCG contents in tea-leaves, as well as higher soil fertility than modern tea plantations, including CEC, SOC, SOM, TN, TP, AN, AP and AK. The long-term plantation in natural cultivation way significantly (*P* < 0.05) improved the abundances of *Nitrospirota* and *Methylomirabilota* phyla as well as 5 genera in bacterial community composition, and the abundances of *Ascomycota* and *Mortierellomycota* phyla as well as 9 genera in fungal community composition. The bivariate correlation analysis and RDA revealed the impact of soil physical properties on tea-leaves chemical components and microbial community composition in tea plantations. The significantly (*P* < 0.05) higher soil fertility promised the stabilization of bacterial abundance and diversity, and improved the abundance and diversity of fungal community. Generally, This study revealed the characteristics of tea-leaves chemical components, soil chemicophysical properties and microbial community structure in the ancient tea plantations, which advances the knowledge about the sustainable development of ancient tea plantations in soil ecosystem.

## Supplementary Information

**Additional file 1: Supplemental Data Sets.** The raw data of tea-leaves quality components, soil environmental factors and microbial community composition in modern and ancient tea plantations of five sampling sites including Bingdao, Baqishan, Banuo, Dongguo and Jiulong, respectively. (XLS 1972 kb)

**Additional file 2: Table S1.** Chemical components in tea-leaves of modern and ancient tea plantations in five sampling sites including Bingdao, Baqishan, Banuo, Dongguo and Jiulong, respectively. **Table S2.** Soil nutrition and microbial population of modern and ancient tea plantations in five sampling sites including Bingdao, Baqishan, Banuo, Dongguo and Jiulong, respectively. **Table S3.** Soil bacterial and fungal community diversity (Chao 1, Shannon and Coverage) of modern and ancient tea plantations in the five sampling sites (i.e. Bingdao, Banuo, Baqishan, Dongguo and Jiulong), respectively. **Table S4.** Bacterial and fungal community diversity (Chao 1, Shannon and Coverage) differences in soil between modern and ancient tea plantations. **Table S5**. Differences of the relative abundance of the top 22 bacterial phyla within soil aggregates between modern and ancient tea plantations. **Table S6**. Differences of the relative abundance of the top 50 bacterial genera within soil aggregates between modern and ancient tea plantations. **Table S7**. Differences of the relative abundance of the top 12 fungal phyla within soil aggregates between modern and ancient tea plantations. **Table S8.** Differences of the relative abundance of the top 50 fungal genera within soil aggregates between modern and ancient tea plantations. **Table S9.** Correlations of soil physical properties to the tea-leaves indicators in tea plantations through the bivariate correlation analysis. **Table S10.** Correlations of physical properties to microbial community abundance and diversity in the soil of tea plantations through the bivariate correlation analysis. **Fig. S1.** The non-metric multidimensional scaling (NMDS) of soil bacterial communities in modern and ancient tea plantations of five various sampling sites (i.e. Bingdao, Banuo, Baqishan, Dongguo and Jiulong), respectively. **Fig. S2.** The non-metric multidimensional scaling (NMDS) of soil fungal communities in modern and ancient tea plantations of five various sampling sites (i.e. Bingdao, Banuo, Baqishan, Dongguo and Jiulong), respectively.

## Data Availability

The plant materials were growing in our resource nursery, which are available from the corresponding author on reasonable request, and deposited in publicly Laboratory of Tea Cultivation and Breeding. All data generated or analyzed during this study are included in this published article and its additional files.

## Abbreviations

CEC: Cation exchange capacity
SOM: Soil organic matter
SOC: Soil organic carbon
TN: Total nitrogen
TP: Total phosphorus
TK: Total potassium
AN: Alkali-hydrolyzable nitrogen
AP: Available phosphorous
AK: Available potassium
HPLC: High performance liquid chromatography
C: Catechin
EC: Epicatechin
EGC: Epigallocatechin
ECG: Epigallocatechin gallate
GCG: Gallocatechin gallate
EGCG: Epigallocatechin gallate
