# Supplementary material for: HPLC and high-throughput sequencing revealed higher tea-leaves quality, soil fertility and microbial community diversity in ancient tea plantations: compared with modern tea plantations
Source: BMC Plant Biol. 2022 May 12;22:239. doi: 10.1186/s12870-022-03633-6 (PMC9097118; doi:10.1186/s12870-022-03633-6)
Supplement: Supplementary file 2 — Additional file 2: Table S1. Chemical components in tea-leaves of modern and ancient tea plantations in five sampling sites including Bingdao, Baqishan, Banuo, Dongguo and Jiulong, respectively. Table S2. Soil nutrition and microbial population of modern and ancient tea plantations in five sampling sites including Bingdao, Baqishan, Banuo, Dongguo and Jiulong, respectively. Table S3. Soil bacterial and fungal community diversity (Chao 1, Shannon and Coverage) of modern and ancient tea plantations in the five sampling sites (i.e. Bingdao, Banuo, Baqishan, Dongguo and Jiulong), respectively. Table S4. Bacterial and fungal community diversity (Chao 1, Shannon and Coverage) differences in soil between modern and ancient tea plantations. Table S5. Differences of the relative abundance of the top 22 bacterial phyla within soil aggregates between modern and ancient tea plantations. Table S6. Differences of the relative abundance of the top 50 bacterial genera within soil aggregates between modern and ancient tea plantations. Table S7. Differences of the relative abundance of the top 12 fungal phyla within soil aggregates between modern and ancient tea plantations. Table S8. Differences of the relative abundance of the top 50 fungal genera within soil aggregates between modern and ancient tea plantations. Table S9. Correlations of soil physical properties to the tea-leaves indicators in tea plantations through the bivariate correlation analysis. Table S10. Correlations of physical properties to microbial community abundance and diversity in the soil of tea plantations through the bivariate correlation analysis. Fig. S1. The non-metric multidimensional scaling (NMDS) of soil bacterial communities in modern and ancient tea plantations of five various sampling sites (i.e. Bingdao, Banuo, Baqishan, Dongguo and Jiulong), respectively. Fig. S2. The non-metric multidimensional scaling (NMDS) of soil fungal communities in modern and ancient tea plantations of five various sampling [file 12870_2022_3633_MOESM2_ESM.doc]

**Table S1.** Chemical components in tea-leaves of modern and ancient tea plantations in five sampling sites including Bingdao, Baqishan, Banuo, Dongguo and Jiulong, respectively.

| Item | Type of tea plantation | Sampling sites | | | | |
| --- | --- | --- | --- | --- | --- | --- |
|  |  | Bingdao | Baqishan | Banuo | Dongguo | Jiulong |
| Water extraction (%) | Modern | 51.40±0.65 | 49.50±1.15 | 52.39±0.83 | 49.93±1.02 | 45.49±1.73 |
|  | Ancient | 52.55±1.19 | 52.13±1.07* | 48.96±0.87*** | 52.15±0.94** | 48.34±1.40* |
| Free amino acids (%) | Modern | 3.17±0.13 | 2.24±0.26 | 2.87±0.16 | 2.77±0.17 | 2.98±0.12 |
|  | Ancient | 3.55±0.21* | 3.01±0.11*** | 3.82±0.15*** | 3.30±0.11*** | 3.47±0.11*** |
| Tea polyphenols (%) | Modern | 28.70±0.54 | 31.55±0.94 | 29.11±1.19 | 28.93±0.75 | 29.04±1.53 |
|  | Ancient | 30.90±0.91** | 33.04±0.92* | 28.47±1.11 | 31.26±1.25** | 30.47±0.91 |
| Polyphenols/Amino acids ratio | Modern | 9.06±0.48 | 14.24±1.65 | 10.15±0.37 | 10.49±0.71 | 9.76±0.72 |
|  | Ancient | 8.72±0.38 | 10.99±0.66** | 7.46±0.16*** | 9.46±0.31* | 8.78±0.37* |
| Caffeine (mg g^-1^) | Modern | 44.51±2.76 | 33.98±0.92 | 42.69±0.52 | 39.04±0.98 | 40.85±1.10 |
|  | Ancient | 45.53±0.61 | 37.52±2.58* | 45.42±0.89*** | 41.49±0.69** | 43.93±1.34** |
| Gallic acid (mg g^-1^) | Modern | 0.28±0.26 | 0.00±0.00 | 0.00±0.00 | 0.15±0.21 | 0.00±0.00 |
|  | Ancient | 0.68±0.08** | 0.50±0.05*** | 0.48±0.06*** | 0.53±0.10** | 0.59±0.11*** |
| C (mg g^-1^) | Modern | 14.03±0.91 | 21.00±1.04 | 7.99±0.27 | 18.98±2.70 | 16.71±3.09 |
|  | Ancient | 18.63±2.21** | 16.87±2.63* | 4.89±0.12*** | 17.24±1.95 | 11.63±1.48* |
| EC (mg g^-1^) | Modern | 26.13±3.69 | 36.83±2.70 | 16.15±0.69 | 32.60±3.73 | 29.97±1.76 |
|  | Ancient | 38.35±1.98*** | 24.10±2.30*** | 13.37±1.27** | 37.38±3.60 | 37.29±2.24*** |
| EGC(mg g^-1^) | Modern | 15.11±2.60 | 22.56±2.19 | 10.19±0.70 | 19.54±2.87 | 23.58±1.85 |
|  | Ancient | 27.56±4.14*** | 32.72±2.56*** | 27.76±1.67*** | 31.23±2.69*** | 33.11±2.27*** |
| ECG (mg g^-1^) | Modern | 103.19±11.07 | 90.50±3.94 | 50.97±6.46 | 92.93±3.85 | 90.72±7.56 |
|  | Ancient | 103.58±6.91 | 65.92±5.07*** | 43.86±1.64 | 83.21±5.27* | 78.64±4.49* |
| GCG (mg g^-1^) | Modern | 5.39±1.55 | 6.55±0.47 | 3.10±0.39 | 6.10±0.77 | 6.10±0.82 |
|  | Ancient | 6.60±1.66 | 3.74±0.65*** | 1.88±0.09** | 5.09±0.62 | 4.42±0.54** |
| EGCG (mg g^-1^) | Modern | 49.04±2.32 | 48.63±1.85 | 51.57±6.34 | 58.73±3.33 | 59.03±6.73 |
|  | Ancient | 42.34±1.09*** | 49.58±7.01 | 40.85±1.64* | 48.22±1.47*** | 43.60±2.34** |
| Total catechins  (mg g^-1^) | Modern | 212.90±15.16 | 226.08±3.36 | 139.97±14.21 | 228.88±3.43 | 226.12±9.87 |
|  | Ancient | 237.06±6.92* | 192.92±10.87** | 132.61±1.99 | 227.38±7.39 | 208.70±7.05* |
| Non-ester catechins (mg g^-1^) | Modern | 55.28±2.20 | 80.39±2.89 | 34.33±1.36 | 71.11±4.98 | 70.26±2.98 |
|  | Ancient | 84.54±5.63*** | 73.69±5.62 | 46.02±2.43*** | 85.85±2.99*** | 82.03±1.77*** |
| Ester catechins  (mg g^-1^) | Modern | 157.62±13.73 | 145.69±4.91 | 105.64±12.91 | 157.76±5.97 | 155.86±10.24 |
|  | Ancient | 152.53±6.92 | 119.24±5.81*** | 86.59±2.00*** | 136.52±5.46*** | 126.67±5.74*** |
| Non-ester catechins/ Ester catechins ratio | Modern | 0.35±0.03 | 0.55±0.03 | 0.33±0.02 | 0.45±0.05 | 0.45±0.04 |
|  | Ancient | 0.56±0.04*** | 0.62±0.03* | 0.53±0.04*** | 0.63±0.02*** | 0.65±0.02*** |

Note: Polyphenols/Amino acids ratio = Tea polyphenols content/ free amino acids content; Total catechins were the summation of C, EC, EGC, ECG, GCG and EGCG contents; Non-ester catechins were the summation of C, EC and EGC contents; Ester catechins were the summation of ECG, GCG and EGCG contents; Non-ester/ Ester catechins ratio =Non-ester catechins content / Ester catechins content. And all data were present by mean value ± standard deviation. Significance difference levels: * *P*<0.05; ** *P*<0.01 and *** *P*<0.001 determined by the independent-samples T-test using SPSS 20.0 for Windows.

**Table S2.** Soil nutrition and microbial population of modern and ancient tea plantations in five sampling sites including Bingdao, Baqishan, Banuo, Dongguo and Jiulong, respectively.

| Item |  | Type of tea plantation | Sampling sites | | | | |
| --- | --- | --- | --- | --- | --- | --- | --- |
|  |  |  | Bingdao | Baqishan | Banuo | Dongguo | Jiulong |
| Soil physical and chemical properties | pH value | Modern | 4.62±0.03 | 4.66±0.01 | 4.77±0.04 | 4.50±0.02 | 4.40±0.02 |
|  |  | Ancient | 4.32±0.03*** | 4.75±0.02*** | 4.64±0.01** | 4.18±0.03*** | 4.45±0.03* |
|  | CEC  (cmol kg^-1^) | Modern | 8.45±0.05 | 6.55±0.04 | 14.62±0.35 | 10.61±0.20 | 5.42±0.08 |
|  |  | Ancient | 10.44±0.04*** | 12.75±0.26*** | 16.73±0.34*** | 12.45±0.07*** | 8.39±0.09*** |
|  | SOC (g kg^-1^) | Modern | 33.89±0.25 | 32.22±0.87 | 46.33±0.60 | 58.07±0.53 | 22.84±2.69 |
|  |  | Ancient | 50.40±0.56*** | 58.82±0.60*** | 49.30±0.52*** | 60.28±0.45*** | 37.76±2.08*** |
|  | SOM (g kg^-1^) | Modern | 58.99±0.89 | 55.79±1.59 | 79.30±0.70 | 99.05±2.03 | 36.53±0.71 |
|  |  | Ancient | 87.26±0.58*** | 99.33±2.71*** | 85.20±0.89*** | 104.24±1.60** | 67.29±0.92*** |
|  | TN (g kg^-1^) | Modern | 1.15±0.07 | 0.84±0.02 | 1.53±0.13 | 0.53±0.01 | 1.88±0.02 |
|  |  | Ancient | 1.54±0.04*** | 1.97±0.04*** | 1.67±0.03 | 0.97±0.01*** | 2.27±0.03*** |
|  | TP (g kg^-1^) | Modern | 0.136±0.004 | 0.293±0.006 | 0.136±0.005 | 0.459±0.003 | 0.260±0.007 |
|  |  | Ancient | 0.536±0.006*** | 0.599±0.023*** | 0.251±0.003*** | 0.711±0.004*** | 0.413±0.011*** |
|  | TK (g kg^-1^) | Modern | 6.14±0.23 | 7.72±0.29 | 5.08±0.01 | 8.12±0.05 | 5.89±0.27 |
|  |  | Ancient | 2.51±0.27*** | 9.08±0.29*** | 7.21±0.32*** | 4.67±0.37*** | 2.56±0.54*** |
|  | AN (mg kg^-1^) | Modern | 134.08±2.15 | 86.34±2.08 | 116.29±2.52 | 105.86±2.90 | 60.16±1.92 |
|  |  | Ancient | 171.07±3.31*** | 144.63±1.64*** | 109.96±2.12** | 148.64±3.19*** | 70.12±0.86*** |
|  | AP (mg kg^-1^) | Modern | 10.98±0.73 | 7.63±0.21 | 16.01±0.16 | 11.90±0.19 | 6.68±0.36 |
|  |  | Ancient | 18.89±0.85*** | 20.72±0.14*** | 20.57±0.88*** | 15.23±0.44*** | 15.57±0.59*** |
|  | AK (mg kg^-1^) | Modern | 117.01±2.63 | 149.82±4.49 | 145.60±1.68 | 116.21±2.47 | 135.16±2.87 |
|  |  | Ancient | 142.83±3.16*** | 227.59±3.49*** | 107.20±4.18*** | 137.71±3.89*** | 142.12±4.18* |
| Soil microbial population | Bacteria  (×10^6^ CFU g^-1^) | Modern | 13.14±0.33 | 30.24±3.04 | 29.17±2.52 | 14.40±0.64 | 17.46±1.96 |
|  |  | Ancient | 24.88±1.35*** | 24.05±1.28** | 18.82±1.77*** | 16.07±1.24* | 17.29±0.99 |
|  | Fungi  (×10^3^ CFU g^-1^) | Modern | 6.70±0.77 | 7.70±0.92 | 7.14±0.53 | 10.60±1.23 | 8.94±0.57 |
|  |  | Ancient | 10.16±0.37*** | 9.79±0.32** | 7.46±0.62 | 17.583±1.44*** | 11.26±0.32*** |
|  | actinomycetes (×10^7^ CFU g^-1^) | Modern | 4.62±0.56 | 6.33±0.770 | 4.55±0.36 | 22.97±2.26 | 10.39±0.72 |
|  |  | Ancient | 17.56±1.61*** | 5.80±0.92 | 5.09±0.47 | 10.15±1.32*** | 12.32±0.30** |

CEC: Cation exchange capacity; SOC: Soil organic carbon; SOM: Soil organic matter; TN: Total nitrogen; TP: Total phosphorus; TK: Total potassium; AN: Alkali-hydrolyzable nitrogen; AP: Available phosphorus; AK: Available potassium.

All data were present by mean value ± standard deviation. Significance difference levels: * *P*<0.05; ** *P*<0.01 and *** *P*<0.001 determined by the independent-samples T-test using SPSS 20.0 for Windows.

**Table S3.** Soil bacterial and fungal community diversity (Chao 1, Shannon and Coverage) of modern and ancient tea plantations in the five sampling sites (i.e. Bingdao, Banuo, Baqishan, Dongguo and Jiulong), respectively.

| Item | | Tea plantation | Sampling sites | | | | |
| --- | --- | --- | --- | --- | --- | --- | --- |
|  |  |  | Bingdao | Baqishan | Banuo | Dongguo | Jiulong |
| **Bacterial community**  **diversity** | Chao 1 | Modern | 3203.27±381.76 | 3290.96±79.78 | 3256.86±271.22 | 3034.47±166.28 | 2754.08±188.95 |
|  |  | Ancient | 3333.97±241.02 | 3046.07±300.60 | 3399.03±242.56 | 3088.74±287.89 | 3215.36±205.97** |
|  | Shannon | Modern | 6.49±0.14 | 6.46±0.12 | 6.52±0.20 | 6.36±0.28 | 6.25±0.38 |
|  |  | Ancient | 6.48±0.18 | 6.20±0.38 | 6.37±0.22 | 6.00±0.55 | 6.46±0.06 |
|  | Coverage (%) | Modern | 97.26±0.48 | 97.08±0.13 | 97.22±0.25 | 97.42±0.20 | 97.70±0.13 |
|  |  | Ancient | 97.12±0.25 | 97.32±0.26 | 96.94±0.21 | 97.19±0.20 | 97.20±0.28* |
| **Fungal community**  **diversity** | Chao 1 | Modern | 954.93±122.12 | 954.65±158.07 | 681.29±108.32 | 708.21±70.92 | 684.35±118.10 |
|  |  | Ancient | 942.68±117.95 | 974.62±214.39 | 958.67±187.36* | 904.49±111.52** | 975.95±100.99** |
|  | Shannon | Modern | 3.96±0.39 | 4.21±0.41 | 3.46±0.83 | 3.69±0.49 | 3.86±0.25 |
|  |  | Ancient | 4.32±0.22 | 4.20±0.64 | 4.22±0.28 | 4.37±0.32* | 4.56±0.22*** |
|  | Coverage (%) | Modern | 99.27±0.09 | 99.23±0.14 | 99.48±0.06 | 99.46±0.46 | 99.49±0.10 |
|  |  | Ancient | 99.36±0.18 | 99.24±0.19 | 99.29±0.16* | 99.35±0.32* | 99.28±0.07** |

Data represent the mean value of five replicates±standard deviation. Significance difference levels: * *P*<0.05; ** *P*<0.01 and *** *P*<0.001 determined by the independent-samples T-test using SPSS 20.0 for Windows.

**Table S4.** Bacterial and fungal community diversity (Chao 1, Shannon and Coverage) differences in soil between modern and ancient tea plantations.

| Item | Modern tea plantations | | Ancient tea plantations | |
| --- | --- | --- | --- | --- |
|  | Mean (n=5*5) | Rang | Mean (n=5*5) | Rang |
| Bacterial Chao 1 | 3107.93±298.12 | 2984.87~3230.99 | 3216.63±273.31 | 3103.82~3329.45 |
| Bacterial Shannon | 6.42±0.24 | 6.32~6.52 | 6.30±0.35 | 6.16~6.45 |
| Bacterial Coverage(%) | 97.34±0.33 | 97.20~97.47 | 97.15±0.25* | 97.05~97.26 |
| Fungal Chao 1 | 796.69±17.09 | 726.13~867.24 | 951.28±14.26** | 892.43~1010.13 |
| Fungal Shannon | 3.83±0.53 | 3.61~4.05 | 4.19±4.48*** | 1.50~1.87 |
| Fungal Coverage (%) | 99.39±0.14 | 99.33~99.44 | 99.30±0.14* | 99.25~99.36 |

Data represent the mean value of five replicates ± standard deviation. Significance difference levels: * *P*<0.05; ** *P*<0.01 and *** *P*<0.001 determined by the independent-samples T-test using SPSS 20.0 for Windows.

**Table S5.** Differences of the relative abundance of the top 22 bacterial phyla within soil aggregates between modern and ancient tea plantations.

| Item | Modern tea plantations | | Ancient tea plantations | |
| --- | --- | --- | --- | --- |
|  | Mean (n=5*5) | Rang | Mean (n=5*5) | Rang |
| WPS-2 | 1.42±1.02 | 1.00~1.84 | 1.28±1.17 | 0.80~1.76 |
| Verrucomicrobiota | 1.64±0.75 | 1.33~1.95 | 1.79±0.82 | 1.46~2.13 |
| Proteobacteria | 25.20±5.41 | 22.97~27.44 | 22.39±7.01 | 19.50~25.28 |
| Planctomycetota | 2.42±1.30 | 1.88~2.95 | 2.55±1.10 | 2.09~3.00 |
| Patescibacteria | 0.742±0.567 | 0.508~0.976 | 0.437±0.255* | 0.332~0.543 |
| Nitrospirota | 0.523±0.387 | 0.364~0.683 | 0.798±0.475* | 0.601~0.994 |
| Myxococcota | 3.23±0.81 | 2.89~3.57 | 3.11±0.85 | 2.76~3.47 |
| Methylomirabilota | 1.20±1.09 | 0.75~1.65 | 2.08±1.22** | 1.58~2.58 |
| Gemmatimonadota | 2.56±0.60 | 2.31~2.81 | 2.52±0.77 | 2.20~2.83 |
| GAL15 | 0.95±1.53 | 0.315~1.578 | 2.10±3.03 | 0.851~3.353 |
| Firmicutes | 2.02±1.81 | 1.28~2.77 | 1.91±2.04 | 1.07~2.75 |
| Chloroflexi | 20.82±7.81 | 17.59~24.04 | 24.94±11.80 | 20.07~29.81 |
| Bacteroidota | 1.68±1.59 | 1.02~2.34 | 0.99±0.68* | 0.71~1.27 |
| Actinobacteriota | 16.57±7.42 | 14.55~17.79 | 13.15±5.08 | 15.68~18.32 |
| Acidobacteriota | 16.17±3.92 | 13.50~19.63 | 17.00±3.20 | 11.05~15.25 |
| Unclassified_k__norank | 0.489±0.234 | 0.392~0.585 | 0.604±0.282 | 0.487~0.720 |
| RCP2-54 | 0.185±0.094 | 0.146~0.224 | 0.228±0.154 | 0.164~0.291 |
| Elusimicrobiota | 0.264±0.156 | 0.199~0.328 | 0.199±0.125 | 0.147~0.250 |
| Desulfobacterota | 0.222±0.235 | 0.125~0.319 | 0.322±0.315 | 0.192~0.452 |
| Cyanobacteria | 0.359±0.507 | 0.150~0.569 | 0.253±0.141 | 0.194~0.311 |
| Bdellovibrionota | 0.340±0.173 | 0.269~0.411 | 0.209±0.087*** | 0.173~0.245 |
| Armatimonadota | 0.218±0.125 | 0.166~0.269 | 0.170±0.114 | 0.123~0.217 |

Data represent the mean value ± standard deviation. Significance difference levels: * *P*<0.05; ** *P*<0.01 and *** *P*<0.001 determined by the independent-samples T-test using SPSS 20.0 for Windows.

**Table S6.** Differences of the relative abundance of the top 50 bacterial genera within soil aggregates between modern and ancient tea plantations.

| Genus ^a^ | Modern tea plantations | Ancient tea plantations |
| --- | --- | --- |
| c__AD3^b^ | 8.634±5.015 | 12.005±8.640 |
| f__Xanthobacteraceae | 4.202±1.668 | 5.620±2.851* |
| o__Acidobacteriales | 4.420±2.129 | 4.375±1.665 |
| o__Subgroup_2 | 2.544±1.998 | 3.043±1.607 |
| o__Vicinamibacterales | 1.972±1.190 | 2.572±1.850 |
| Bradyrhizobium^a^ | 2.365±0.740 | 2.222±0.844 |
| o__IMCC26256 | 1.986±0.657 | 2.031±0.700 |
| f__Gemmataceae | 1.882±1.189 | 2.065±0.989 |
| o__Elsterales | 1.633±0.794 | 1.769±1.215 |
| c__TK10 | 1.570±0.607 | 2.010±0.895* |
| f__Gemmatimonadaceae | 1.642±0.501 | 1.888±0.725 |
| HSB_OF53-F07 | 1.132±0.735 | 2.050±1.874* |
| o__Gaiellales | 1.528±1.447 | 1.406±1.277 |
| o__Rokubacteriales | 1.137±1.040 | 1.975±1.129** |
| Candidatus_Solibacter | 1.353±0.570 | 1.292±0.533 |
| p__GAL15 | 0.946±1.529 | 2.102±3.030 |
| Bryobacter | 1.430±0.536 | 1.178±0.546 |
| Acidibacter | 1.500±0.790 | 1.242±0.573 |
| p__WPS-2 | 1.419±1.024 | 1.280±1.167 |
| f__JG30-KF-AS9 | 1.148±0.741 | 1.143±0.679 |
| g__unclassified_f__Micrococcaceae | 1.547±1.811 | 1.065±0.790 |
| Acidothermus | 1.020±0.498 | 1.125±0.597 |
| g__unclassified_o__Acidobacteriales | 0.990±0.705 | 1.194±0.732 |
| o__B12-WMSP1 | 1.110±1.054 | 0.956±1.075 |
| Conexibacter | 1.098±0.604 | 0.908±0.406 |
| Anaeromyxobacter | 1.031±0.527 | 1.183±0.585 |
| g__unclassified_f__Ktedonobacteraceae | 0.928±0.738 | 0.955±1.165 |
| Candidatus_Udaeobacter | 0.787±0.653 | 1.052±0.656 |
| MND1 | 0.888±0.391 | 0.759±0.336 |
| Bacillus | 0.843±0.876 | 0.979±1.696 |
| Sphingomonas | 1.095±0.856 | 0.604±0.573* |
| Burkholderia-Caballeronia-Paraburkholderia | 1.027±0.600 | 0.522±0.291*** |
| o__Subgroup_7 | 0.692±0.382 | 0.750±0.332 |
| Mycobacterium | 0.853±0.386 | 0.583±0.332* |
| Haliangium | 0.719±0.273 | 0.560±0.247* |
| Nitrospira | 0.520±0.385 | 0.794±0.473* |
| Gemmatimonas | 0.706±0.392 | 0.501±0.320* |
| f__Acidobacteriaceae_Subgroup_1 | 0.685±0.450 | 0.487±0.432 |
| f__Xanthobacteraceae | 0.536±0.266 | 0.513±0.245 |
| c__JG30-KF-CM66 | 0.537±0.160 | 0.506±0.184 |
| c__KD4-96 | 0.603±0.642 | 0.535±0.548 |
| Kitasatospora | 0.652±0.353 | 0.417±0.148** |
| Ellin6067 | 0.596±0.326 | 0.431±0.279 |
| k__norank_d__Bacteria | 0.489±0.234 | 0.604±0.282 |
| JG30a-KF-32 | 0.441±0.338 | 0.582±0.366 |
| Pedomicrobium | 0.423±0.302 | 0.627±0.512 |
| FCPS473 | 0.471±0.311 | 0.538±0.456 |
| p__Latescibacterota | 0.393±0.506 | 0.573±0.588 |
| f__Micropepsaceae | 0.523±0.217 | 0.424±0.326 |
| f__Ktedonobacteraceae | 0.389±0.278 | 0.353±0.306 |

Note: ^a^ represent the assigned genus,

^b^ represent the no rank or unclassified genus showing the last family, order, class, phylum, kingdom or domain.

Data represent the mean value ± standard deviation. Significance difference levels: * *P*<0.05; ** *P*<0.01 and *** *P*<0.001 determined by the independent-samples T-test using SPSS 20.0 for Windows.

**Table S7.** Differences of the relative abundance of the top 12 fungal phyla within soil aggregates between modern and ancient tea plantations.

| Item | Modern tea plantations | | Ancient tea plantations | |
| --- | --- | --- | --- | --- |
|  | Mean (n=5*5) | Rang | Mean (n=5*5) | Rang |
| Kickxellomycota | 0.028±0.030 | 0.016~0.041 | 0.128±0.260 | 0.021~0.236 |
| unclassified_k__Fungi | 15.549±15.994 | 8.947~22.151 | 7.573±9.836* | 3.513~11.633 |
| Glomeromycota | 0.609±0.706 | 0.318~0.901 | 0.390±0.383 | 0.232~0.548 |
| Ascomycota | 56.217±11.420 | 51.503~60.931 | 65.749±10.980** | 61.217~70.281 |
| Mucoromycota | 0.297±0.706 | 0.006~0.589 | 0.171±0.265 | 0.062~0.280 |
| Zoopagomycota | 0.0039±0.012 | 0.00~0.0087 | 0.0198±0.0755 | 0.00~0.051 |
| Rozellomycota | 6.292±8.308 | 2.863~9.721 | 1.610±2.634* | 0.522~2.697 |
| Mortierellomycota | 3.569±2.683 | 2.458~4.681 | 5.080±2.830* | 3.912~6.248 |
| Basidiomycota | 17.063±10.355 | 12.789~21.337 | 19.016±9.165 | 15.232~22.799 |
| Calcarisporiellomycota | 0.144±0.404 | 0.00~0.311 | 0.136±0.256 | 0.031~0.242 |
| Chytridiomycota | 0.188±0.256 | 0.082~0.294 | 0.097±0.104 | 0.054~0.140 |
| Monoblepharomycota | 0.014±0.017 | 0.007~0.021 | 0.010±0.013 | 0.005~0.016 |

Data represent the mean value ± standard deviation. Significance levels: * *P*<0.05; ** *P*<0.01 and *** *P*<0.001 determined by the independent-samples T-test using SPSS 20.0 for Windows.

**Table S8.** Differences of the relative abundance of the top 50 fungal genera within soil aggregates between modern and ancient tea plantations.

| Genus ^a^ | Modern tea plantations | Ancient tea plantations |
| --- | --- | --- |
| unclassified_k__Fungi ^b^ | 15.549±15.994 | 7.573±9.836* |
| Saitozyma | 7.322±3.988 | 11.166±5.575** |
| p__Ascomycota | 8.507±7.198 | 11.156±6.874 |
| Mortierella | 3.443±2.565 | 5.018±2.870* |
| p__Rozellomycota | 6.182±8.302 | 1.516±2.661* |
| Penicillium | 3.387±3.237 | 3.327±3.033 |
| Chaetomium | 2.859±1.642 | 4.318±5.794 |
| c__Eurotiomycetes | 3.830±6.130 | 2.890±3.502 |
| o__Chaetothyriales | 2.811±2.350 | 3.308±2.950 |
| Fusarium | 2.857±2.742 | 1.818±1.558 |
| f__Didymellaceae | 2.385±6.499 | 2.298±3.124 |
| f__Chaetomiaceae | 1.408±1.782 | 1.038±0.866 |
| Metarhizium | 0.859±0.796 | 1.533±1.443* |
| f__Clavariaceae | 0.348±1.219 | 2.188±6.670* |
| o__Trechisporales | 1.976±5.281 | 0.092±0.225 |
| Staphylotrichum | 0.700±0.945 | 1.399±2.029 |
| c__Archaeorhizomycetes | 0.999±2.392 | 0.946±2.029 |
| f__Chaetosphaeriaceae | 0.975±1.112 | 0.671±0.693 |
| c__Agaricomycetes | 0.948±2.199 | 0.922±3.325 |
| Laccaria | 1.659±5.518 | 0.000±0.009 |
| Hygrocybe | 0.513±1.661 | 1.133±3.639 |
| c__Sordariomycetes | 0.696±1.288 | 0.788±0.737 |
| Pseudogymnoascus | 0.437±0.486 | 0.938±1.020* |
| Coniochaeta | 1.100±2.162 | 0.323±0.290 |
| Purpureocillium | 0.454±0.458 | 0.945±1.351 |
| Aspergillus | 1.181±3.638 | 0.219±0.411 |
| o__Pleosporales | 0.456±0.489 | 0.710±0.623 |
| Trichoderma | 0.671±1.238 | 0.652±0.867 |
| Solicoccozyma | 0.533±0.784 | 0.718±0.862 |
| o__Helotiales | 0.405±0.317 | 0.829±1.021* |
| Leohumicola | 0.438±0.741 | 0.814±0.778* |
| Tolypocladium | 0.152±0.282 | 0.882±1.033*** |
| c__Leotiomycetes | 0.619±0.902 | 0.617±1.383 |
| f__Nectriaceae | 0.312±0.607 | 0.671±0.916 |
| Cephalotrichum | 0.925±4.463 | 0.038±0.068 |
| Thelonectria | 0.351±0.603 | 0.498±0.703 |
| Phialocephala | 0.544±0.702 | 0.248±0.165* |
| Archaeorhizomyces | 0.133±0.276 | 0.743±2.713 |
| Trichoglossum | 0.002±0.007 | 0.752±2.594** |
| Cladosporium | 0.293±0.370 | 0.488±0.682 |
| f__Cordycipitaceae | 0.277±0.967 | 0.514±1.748 |
| Neophaeococcomyces | 0.350±0.469 | 0.415±0.874 |
| Apiotrichum | 0.224±0.742 | 0.511±1.064 |
| Trichocladium | 0.149±0.260 | 0.514±1.708 |
| Talaromyces | 0.344±0.275 | 0.253±0.311 |
| f__Helotiales_fam_Incertae_sedis | 0.301±0.387 | 0.352±0.474 |
| Mycena | 0.383±1.313 | 0.261±0.637 |
| Exophiala | 0.235±0.251 | 0.407±0.422 |
| f__Leotiaceae | 0.170±0.279 | 0.494±1.946 |
| Ciliophora | 0.233±0.382 | 0.362±0.465 |

Note: ^a^ represent the assigned genus,

^b^ represent the no rank or unclassified genus showing the last family, order, class, phylum, kingdom or domain.

Data represent the mean value ± standard deviation. Significance levels: * *P*<0.05; ** *P*<0.01 and *** *P*<0.001 determined by the independent-samples T-test using SPSS 20.0 for Windows.

Table S9. Correlations of soil physical properties to the tea-leaves indicators in tea plantations through the bivariate correlation analysis.

| **Items** | **Soil physical properties** | | | | | | | | | |
| --- | --- | --- | --- | --- | --- | --- | --- | --- | --- | --- |
| **Tea-leaves indicators** | **pH** | **CEC** | **SOC** | **SOM** | **TN** | **TP** | **TK** | **AN** | **AP** | **AK** |
| Water extraction | -0.139 | 0.190 | 0.219 | 0.221 | -0.514** | 0.105 | -0.094 | 0.483** | 0.155 | -0.108 |
| Free amino acids | -0.294* | -0.023 | 0.351* | 0.323* | 0.472** | 0.577** | -0.191 | 0.446** | 0.405** | 0.267 |
| Tea polyphenols | -0.120 | 0.494** | 0.191 | 0.204 | 0.096 | -0.060 | -0.432** | 0.154 | 0.448** | -0.179 |
| Polyphenols/Amino acids ratio | 0.236 | 0.244 | -0.182 | -0.152 | -0.428** | -0.498** | 0.094 | -0.318* | -0.179 | -0.324* |
| Caffeine | -0.143 | -0.364** | 0.008 | -0.008 | 0.255 | 0.386** | -0.096 | 0.307* | 0.091 | 0.410** |
| Gallic acid | -0.426** | 0.234 | 0.460** | 0.446** | 0.355* | 0.486** | -0.445** | 0.478** | 0.601** | 0.008 |
| C | -0.237 | 0.294* | 0.160 | 0.182 | -0.268 | -0.189 | -0.329* | 0.133 | 0.039 | -0.406** |
| EC | -0.552** | 0.052 | 0.186 | 0.216 | -0.024 | 0.125 | -0.795** | 0.140 | 0.041 | -0.067 |
| EGC | -0.318* | 0.428** | 0.453** | 0.433** | 0.564** | 0.399** | -0.383** | 0.179 | 0.624** | -0.061 |
| ECG | -0.418** | -0.187 | -0.099 | -0.074 | -0.269 | -0.221 | -0.451** | 0.176 | -0.258 | -0.366** |
| GCG | -0.392** | -0.075 | -0.051 | -0.030 | -0.215 | -0.182 | -0.463** | 0.067 | -0.225 | -0.216 |
| EGCG | 0.086 | -0.235 | -0.295* | -0.268 | -0.451** | -0.322* | 0.240 | -0.471** | -0.611** | -0.450** |
| Total catechins | -0.512** | -0.040 | 0.113 | 0.145 | -0.235 | 0.013 | -0.497** | 0.166 | -0.137 | -0.268 |
| Non-ester catechins | -0.563** | 0.273 | 0.352* | 0.368** | 0.116 | 0.231 | -0.779** | 0.243 | 0.279 | -0.128 |
| Ester catechins | -0.420** | -0.253 | -0.139 | -0.106 | -0.320* | -0.226 | -0.316* | 0.023 | -0.396** | -0.425** |
| Non-ester/ Ester catechins ratio | -0.278 | 0.487** | 0.456** | 0.447** | 0.466** | 0.318* | -0.419** | 0.168 | 0.607** | -0.039 |

CEC: Cation exchange capacity; SOC: Soil organic carbon; SOM: Soil organic matter; TN: Total nitrogen; TP: Total phosphorus; TK: Total potassium; AN: Alkali-hydrolyzable nitrogen; AP: Available phosphorus; AK: Available potassium.

The bivariate correlation analysis was carried out to obtain the Spearman correlation coefficient. Significance correlation levels: * *P*<0.05; ** *P*<0.01.

**Table S10.** Correlations of physical properties to microbial community abundance and diversity in the soil of tea plantations through the bivariate correlation analysis.

| **Items** | | **Soil physical properties** | | | | | | | | | |
| --- | --- | --- | --- | --- | --- | --- | --- | --- | --- | --- | --- |
|  |  | **pH** | **CEC** | **SOC** | **SOM** | **TN** | **TP** | **TK** | **AN** | **AP** | **AK** |
| Microbial population | Bacteria | 0.403** | 0.114 | -0.113 | -0.128 | 0.148 | 0.013 | -0.011 | 0.068 | 0.266 | 0.690** |
|  | Actinomycetes | -0.672** | -0.326* | 0.237 | 0.257 | -0.115 | 0.602** | -0.253 | -0.106 | -0.136 | -0.059 |
|  | Fungi | -0.633** | -0.039 | 0.549** | 0.550** | 0.066 | 0.823** | -0.306* | 0.162 | 0.120 | 0.107 |
| Bacterial diversity | Shannon | 0.154 | 0.020 | -0.118 | -0.123 | -0.087 | -0.226* | -0.107 | 0.087 | 0.038 | 0.081 |
|  | Chao 1 | 0.163 | 0.214 | 0.045 | 0.051 | -0.097 | -0.106 | -0.149 | 0.201 | 0.302* | 0.082 |
| Fungal  diversity | Shannon | -0.184 | 0.019 | 0.201 | 0.154 | 0.234 | 0.331* | -0.247 | 0.146 | 0.282* | 0.113 |
|  | Chao 1 | -0.035 | -0.019 | 0.090 | 0.056 | 0.154 | 0.174 | -0.095 | 0.241 | 0.254 | 0.0127 |

CEC: Cation exchange capacity; SOC: Soil organic carbon; SOM: Soil organic matter; TN: Total nitrogen; TP: Total phosphorus; TK: Total potassium; AN: Alkali-hydrolyzable nitrogen; AP: Available phosphorus; AK: Available potassium.

The bivariate correlation analysis was carried out to obtain the Spearman correlation coefficient. Significance correlation levels: * *P*<0.05; ** *P*<0.01.


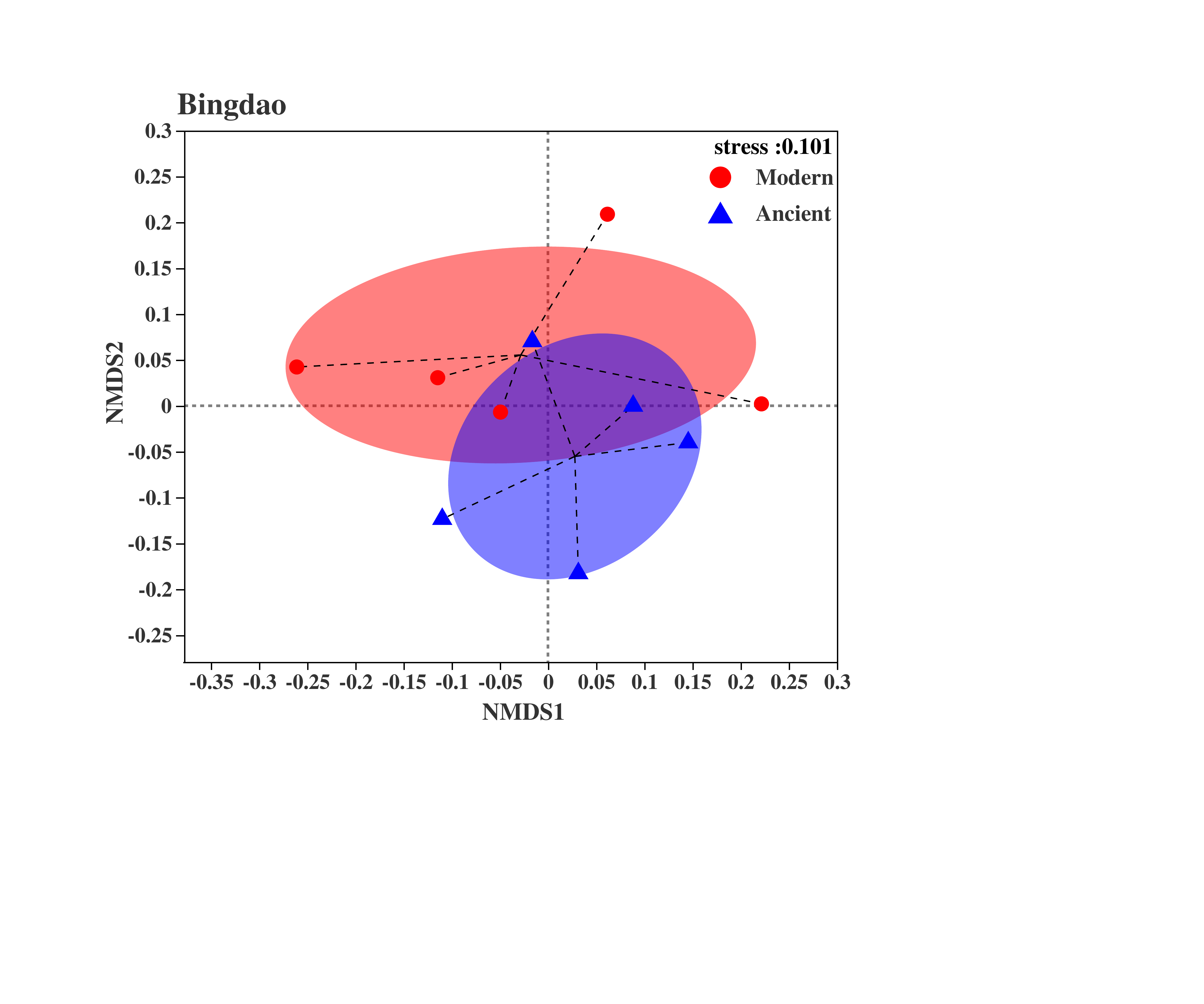

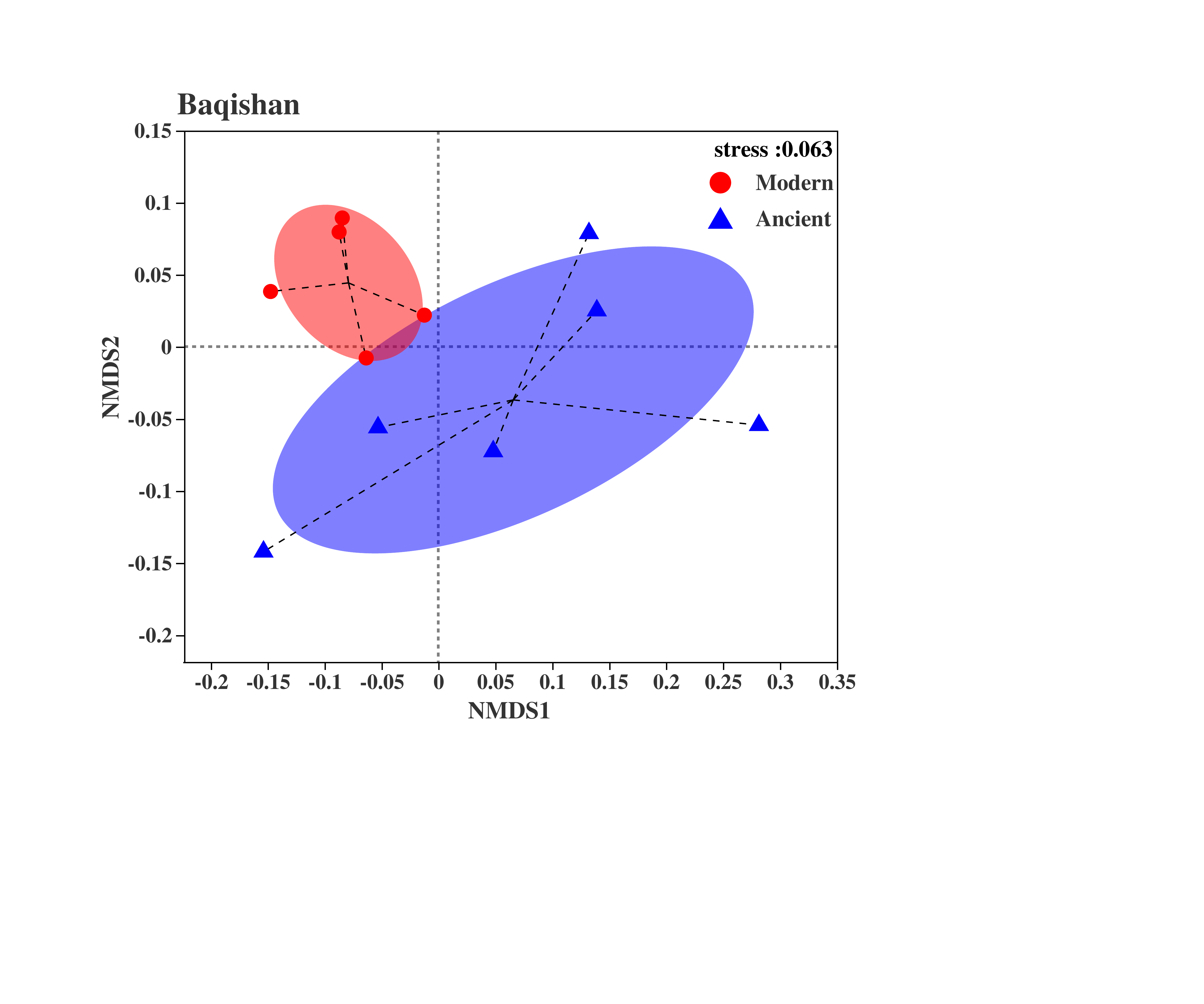

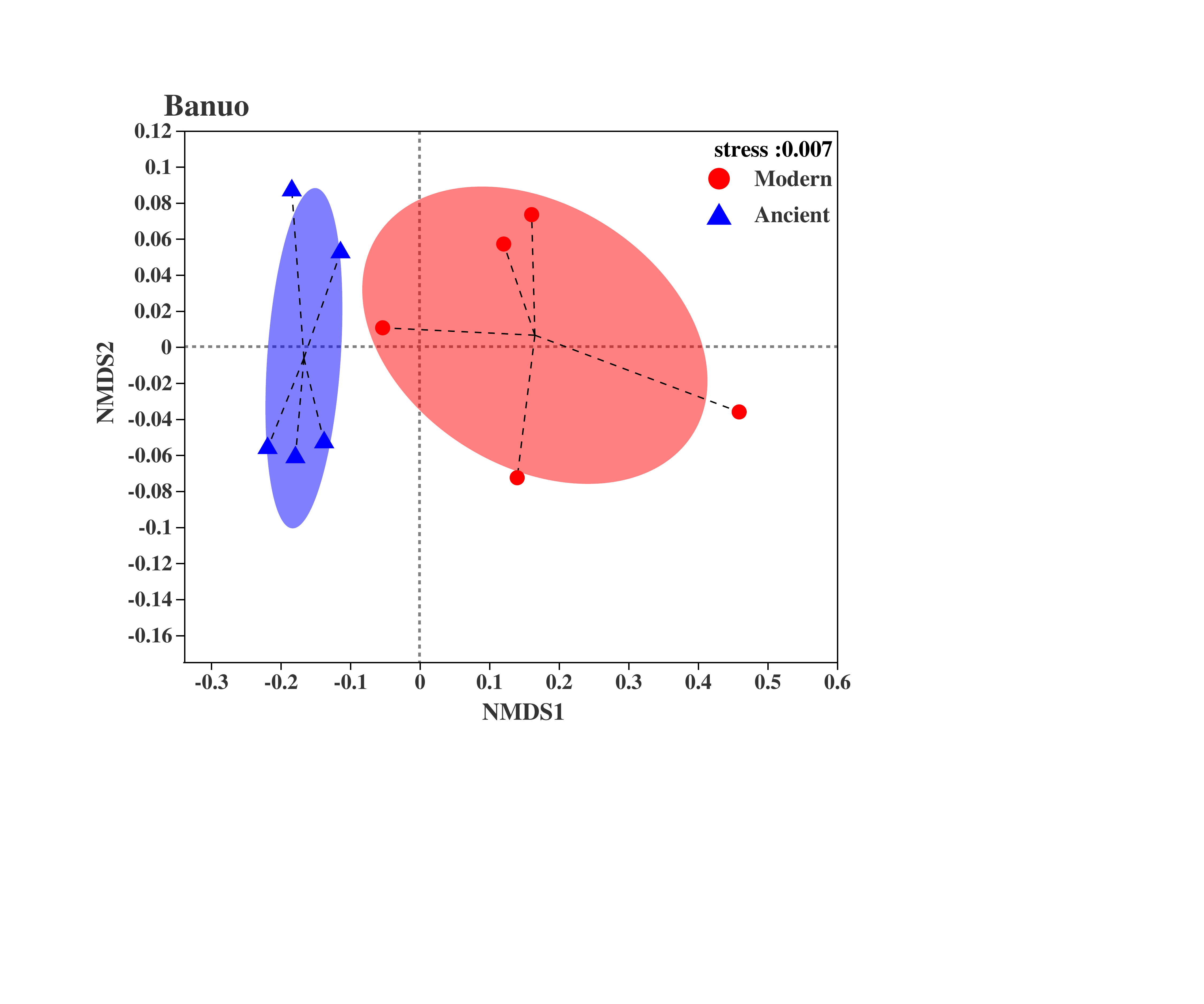


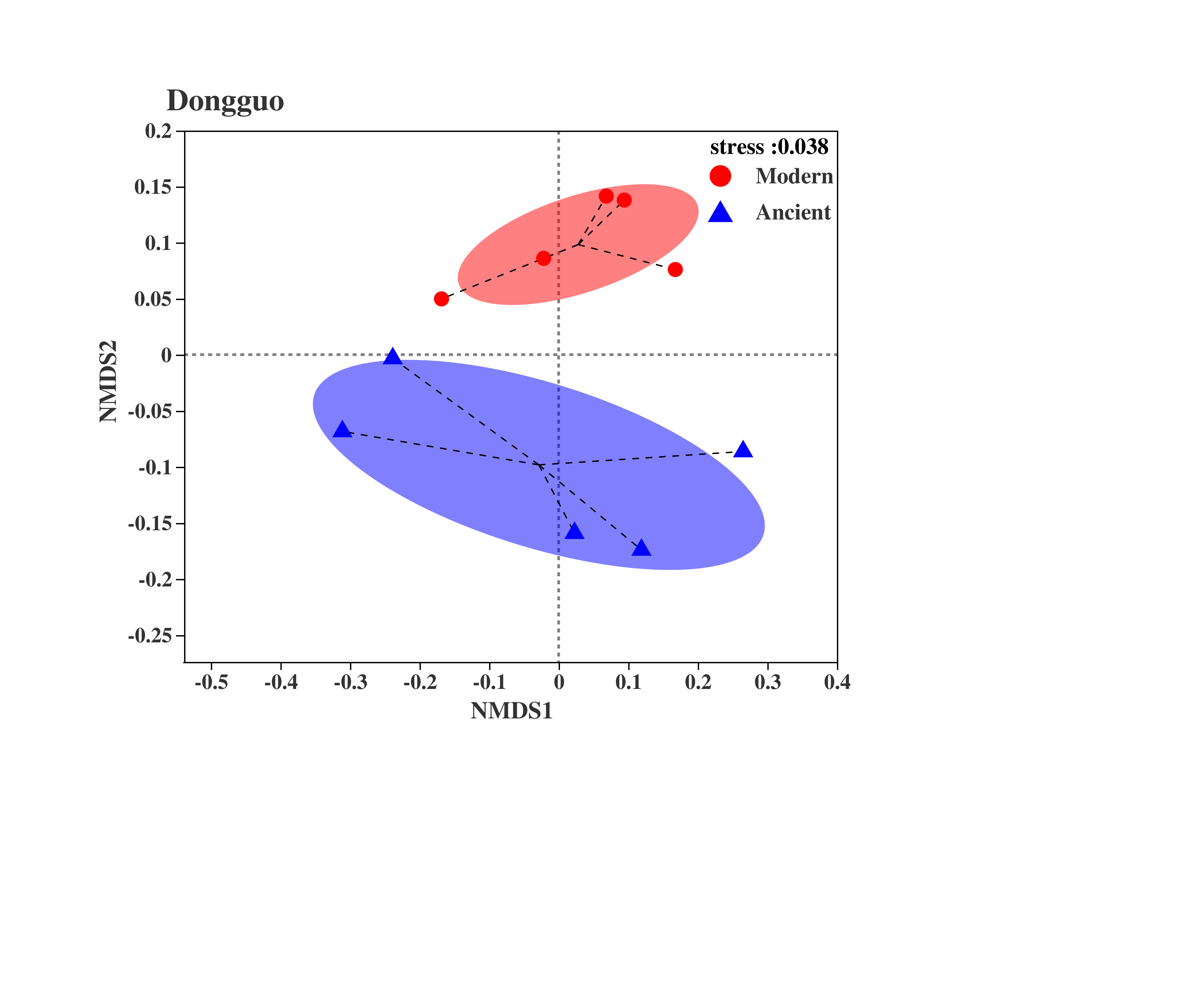

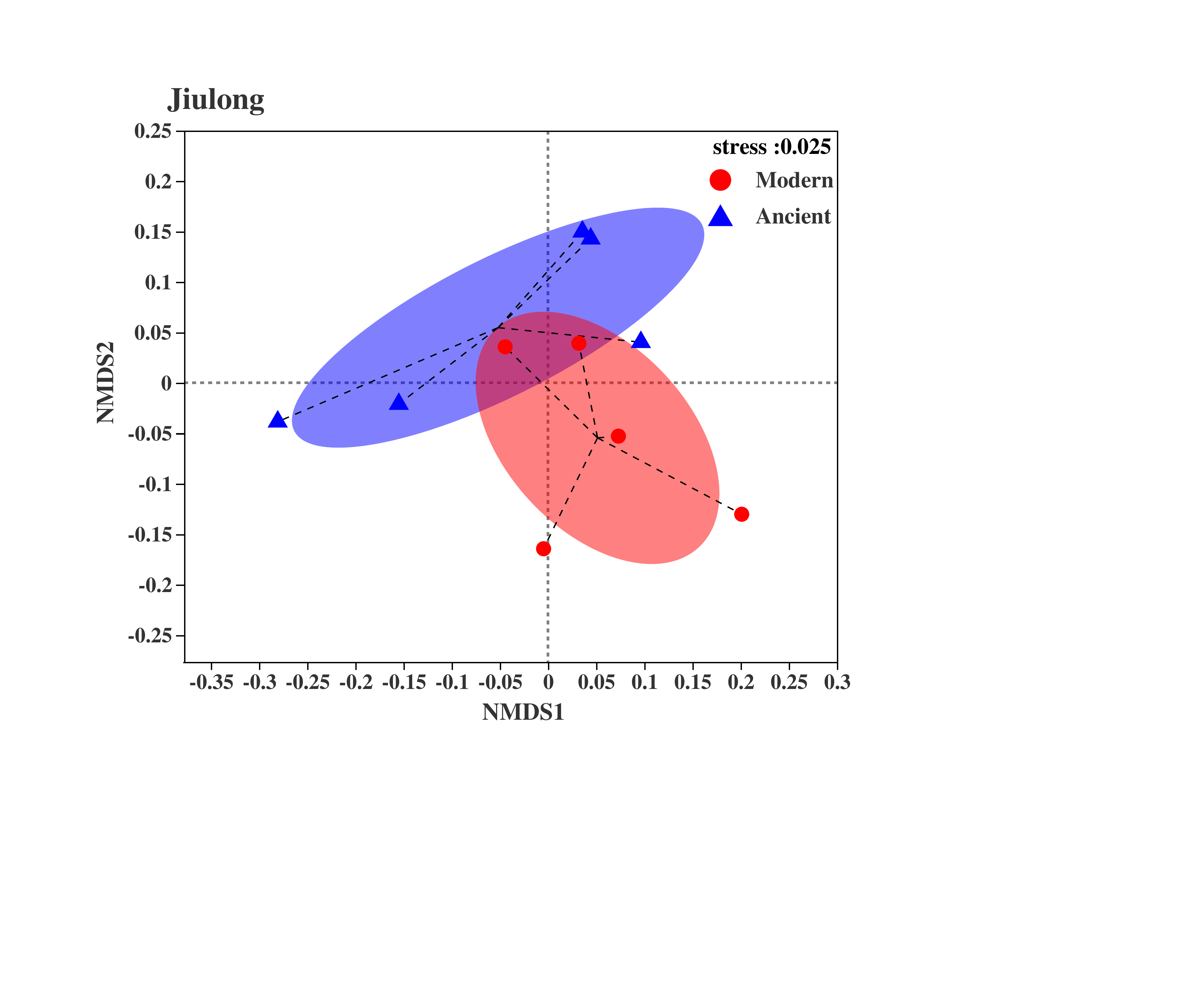


**Fig. S1.** The non-metric multidimensional scaling (NMDS) of soil bacterial communities in modern and ancient tea plantations of five various sampling sites (i.e. Bingdao, Banuo, Baqishan, Dongguo and Jiulong), respectively.


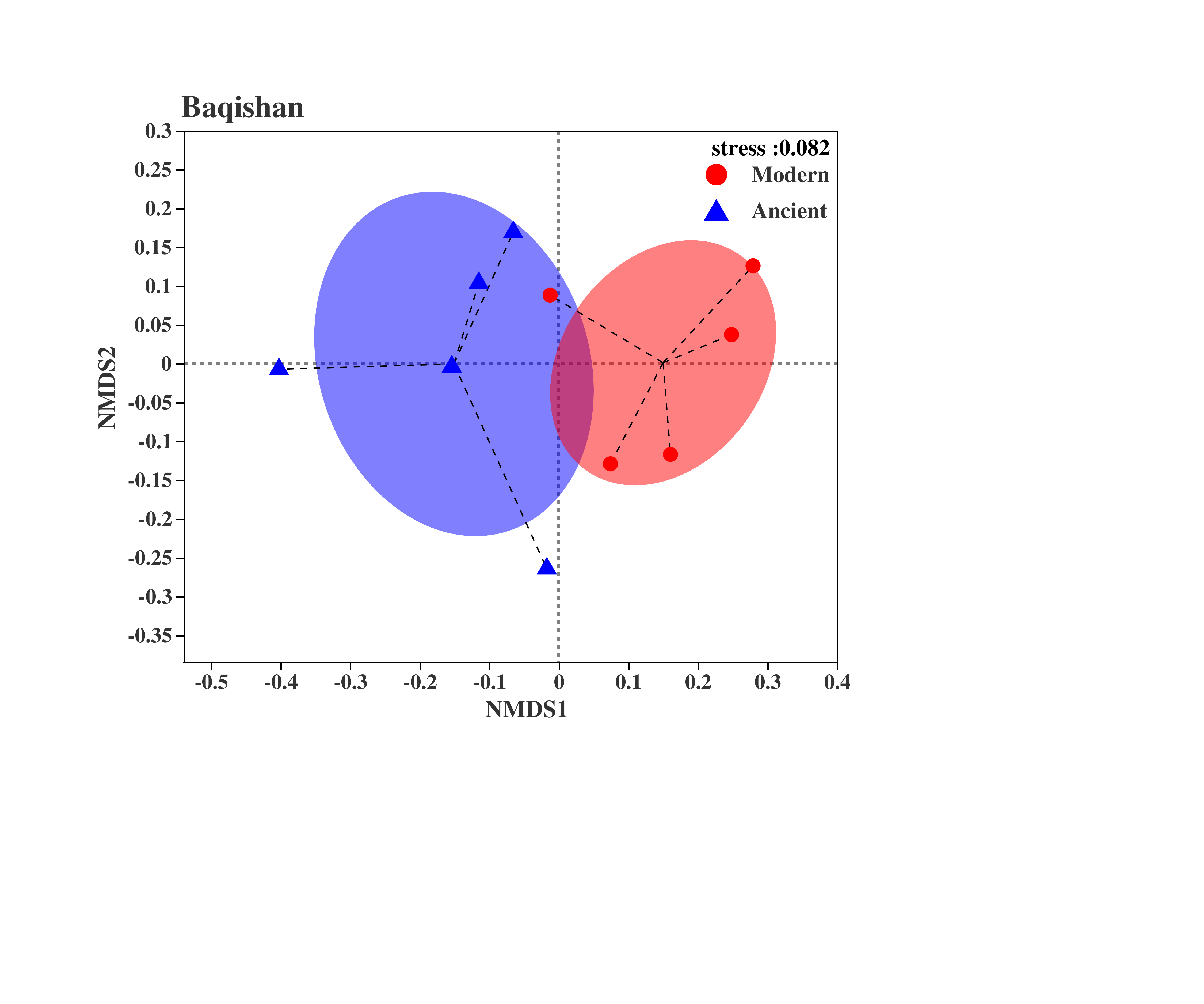

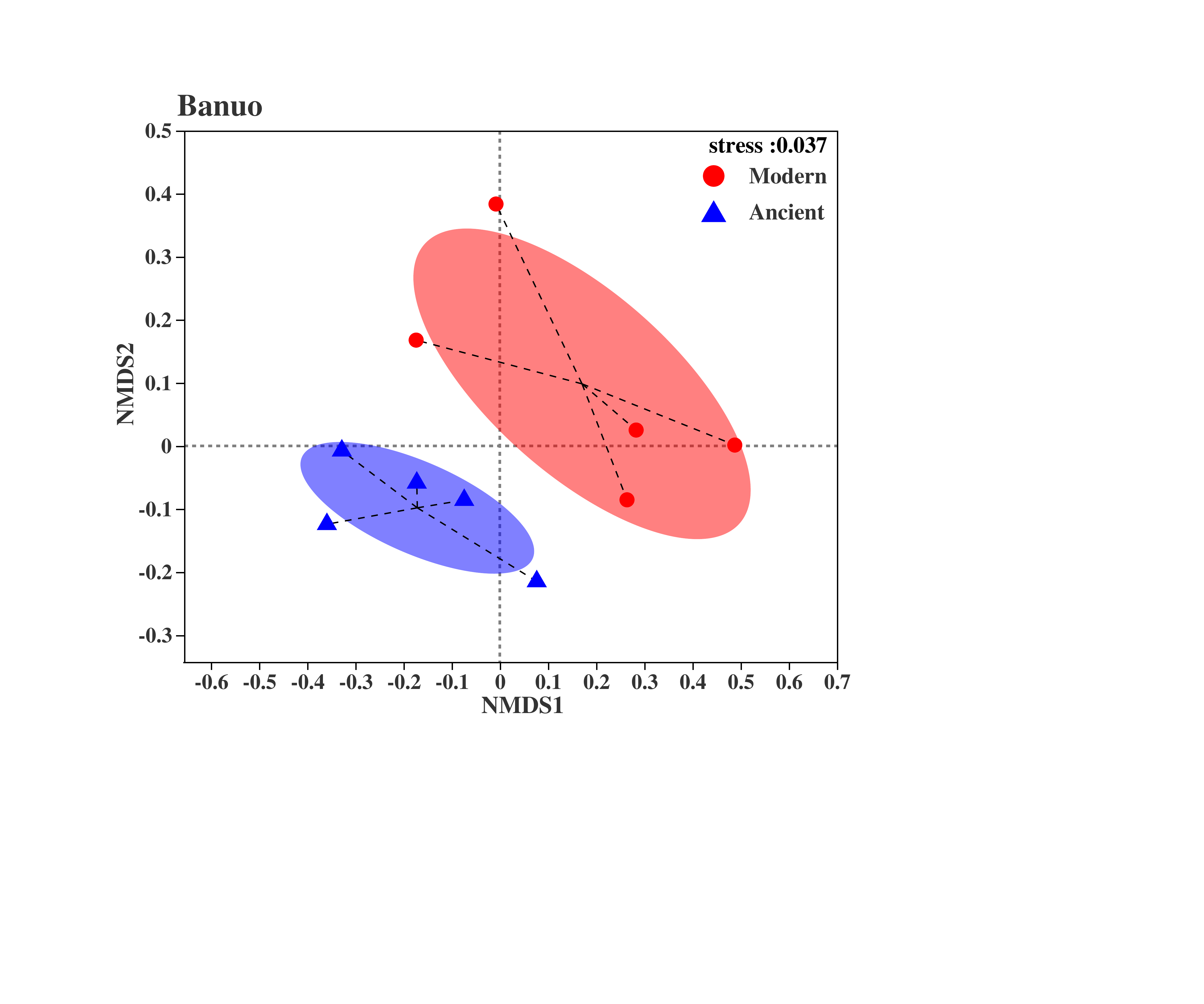

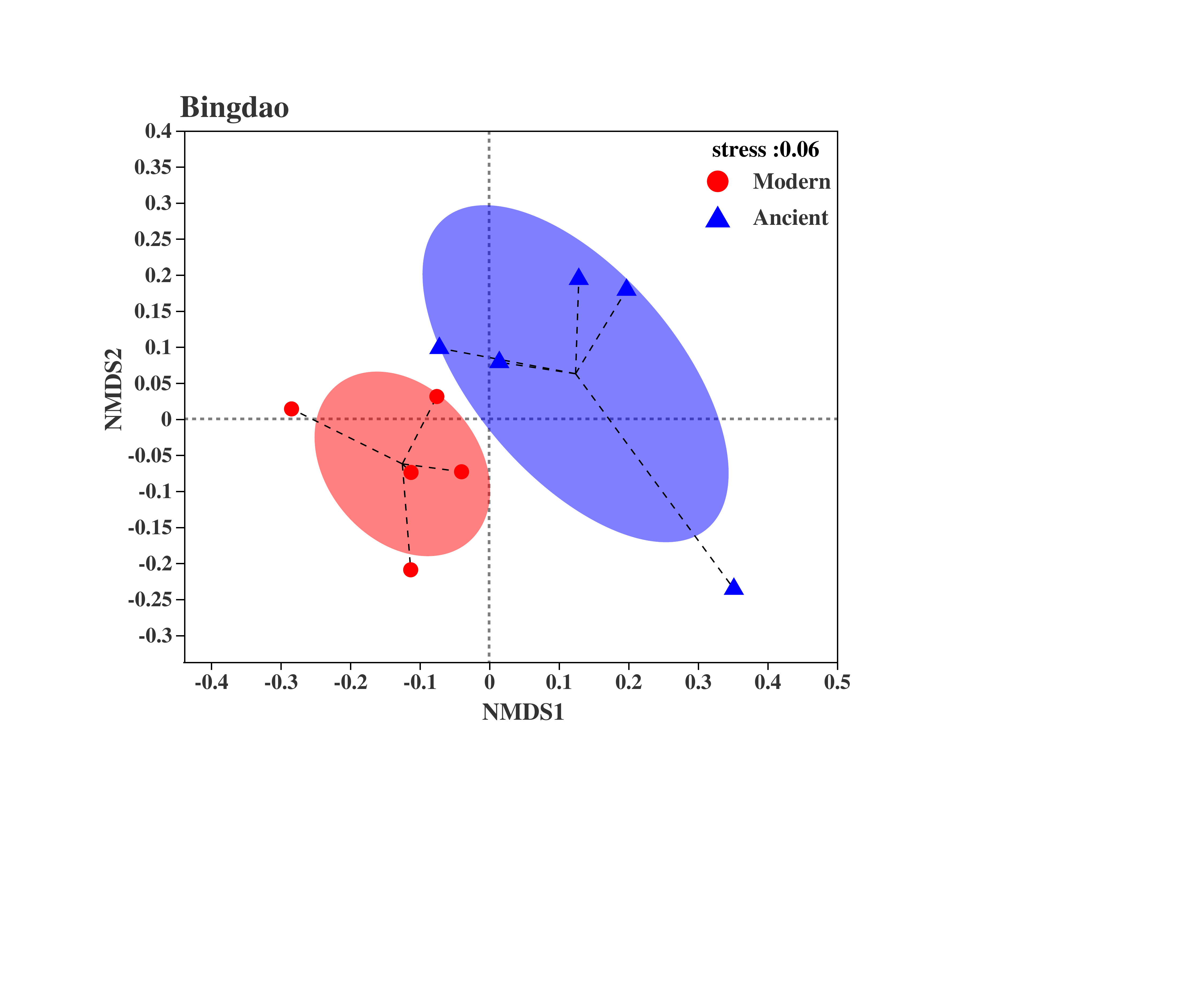


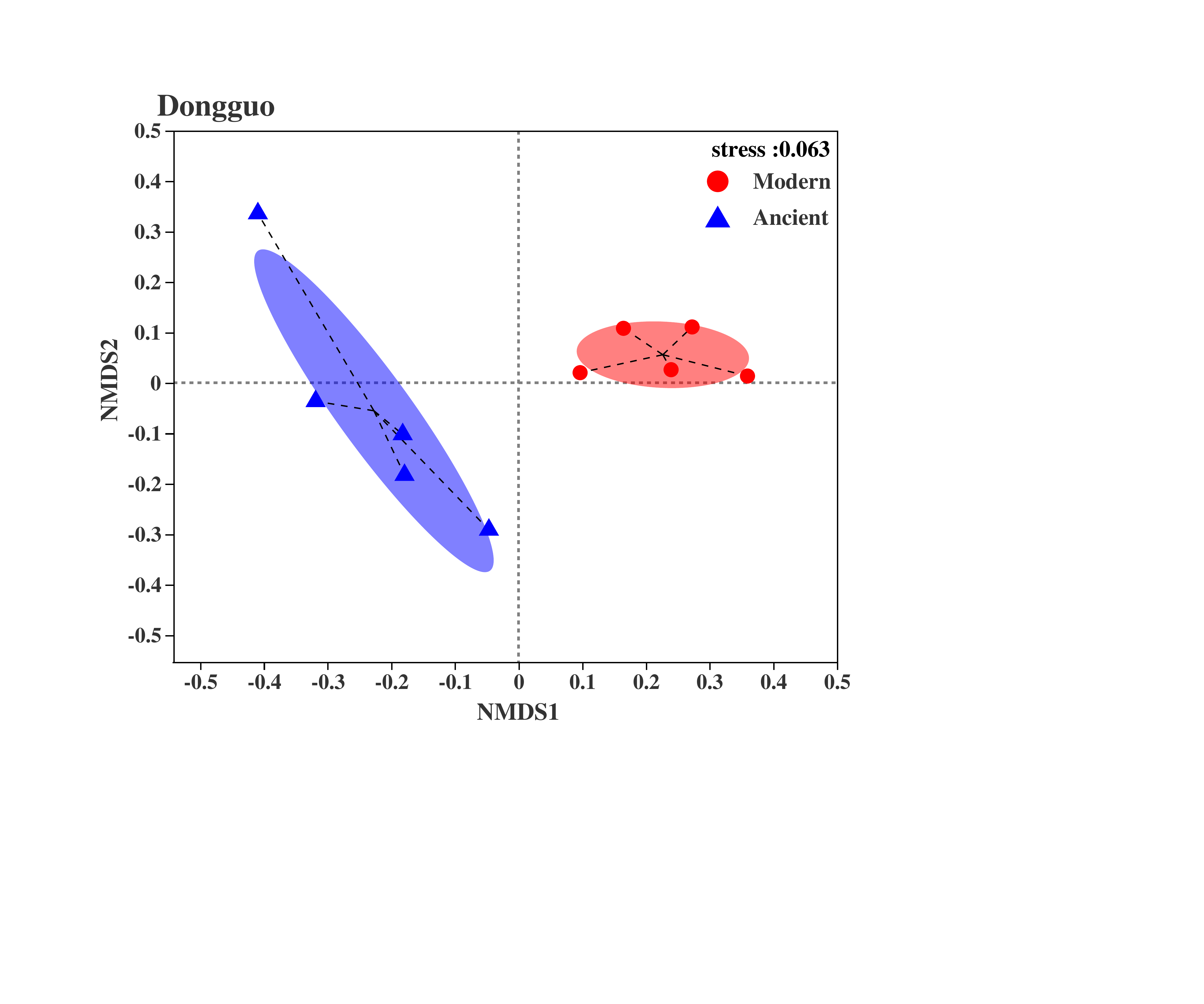

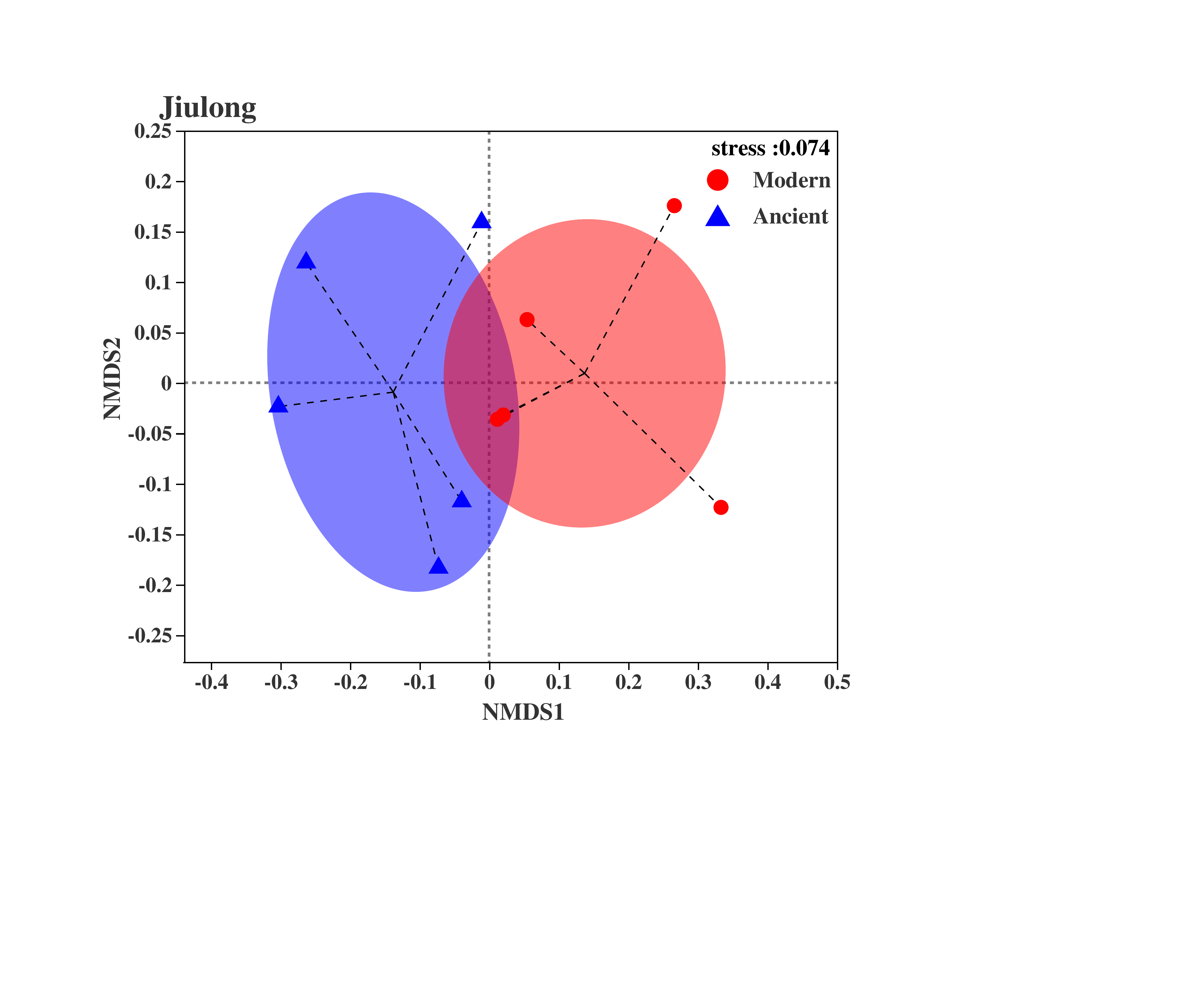


**Fig. S2.** The non-metric multidimensional scaling (NMDS) of soil fungal communities in modern and ancient tea plantations of five various sampling sites (i.e. Bingdao, Banuo, Baqishan, Dongguo and Jiulong), respectively.
